# Forelimb motion and reciprocation mediate aerodynamic control in a gliding lizard

**DOI:** 10.1186/s12862-025-02419-2

**Published:** 2025-11-06

**Authors:** Erik A. Sathe, Robert Dudley

**Affiliations:** 1 Department of Integrative Biology, University of California, Berkeley, Berkeley USA; 2 Museum of Vertebrate Zoology, Berkeley, USA; 3 Smithsonian Tropical Research Institute, Panama, Republic of Panama

**Keywords:** Aerial origins, Biomechanics, Flight evolution, Gliding

## Abstract

**Background:**

The origin of the flight stroke in vertebrate flight evolution remains obscure. However, using forelimbs to control aerodynamic forces while gliding provides a possible exaptation from which wingless taxa evolved incipient wing flapping and powered flight. We used flat-tailed house geckos (*Hemidactylus platyurus*) to model the possible dynamics of those gliding taxa ancestral to vertebrate flyers, and characterized their limb and body kinematics while gliding in a vertical wind tunnel, so as to determine biomechanical consequences of forelimb movements during controlled aerial behavior.

**Results:**

Geckos mostly assumed a stereotypical skydiving posture but intermittently would flex the body ventrally as the forelimbs were retracted posteriorly. Shoulder retraction, spinal column flexion, and subsequent translational velocity in the vertical and cranial directions were positively correlated; such alteration of body posture with simultaneous forelimb displacement thus modulates the directions and magnitudes of aerodynamic forces, including horizontal thrust production. Independent of shoulder retraction and body bend, body pitch correlated positively with vertical acceleration and negatively with horizontal acceleration.

**Conclusions:**

Gliding geckos actively use their forelimbs to alter body speed and to generate thrust, suggesting aerodynamic function for limb displacement and reciprocation in the absence of wings. Prior to the origin of the flapping of winglike structures, analogous forelimb motions (including symmetric reciprocation) may have thus provided biomechanical advantage in the evolution of volant vertebrates.

**Supplementary Information:**

The online version contains supplementary material available at 10.1186/s12862-025-02419-2.

## Background

Although the origins of wing flapping in birds, bats, and pterosaurs remain unresolved paleontologically, control of gliding and aerial maneuvering via forelimb motions provides a biomechanical pathway for flight acquisition [1–3]. In such cases, gliding is powered by gravity while horizontal lift-based forces on mobile axial and appendicular structures can be used to control the flight trajectory [4]. However, the functionality of forelimb displacement prior to sustained flapping is unclear, especially for taxa with only rudimentary wings [2, 5–7]. Theoretical models suggest that rudimentary wing flapping can increase both vertical and horizontal forces [1, 6], whereas studies on physical models [2, 8, 9] and other extant taxa [10–15] have demonstrated aerodynamic consequences of static limb postures and rudimentary flapping for pre-volant morphologies and non-volant gliding morphologies, respectively. Nonetheless, the effects of active forelimb displacement and reciprocation have not been evaluated in non-volant, yet aerially capable tetrapods.

**Fig. 1:**
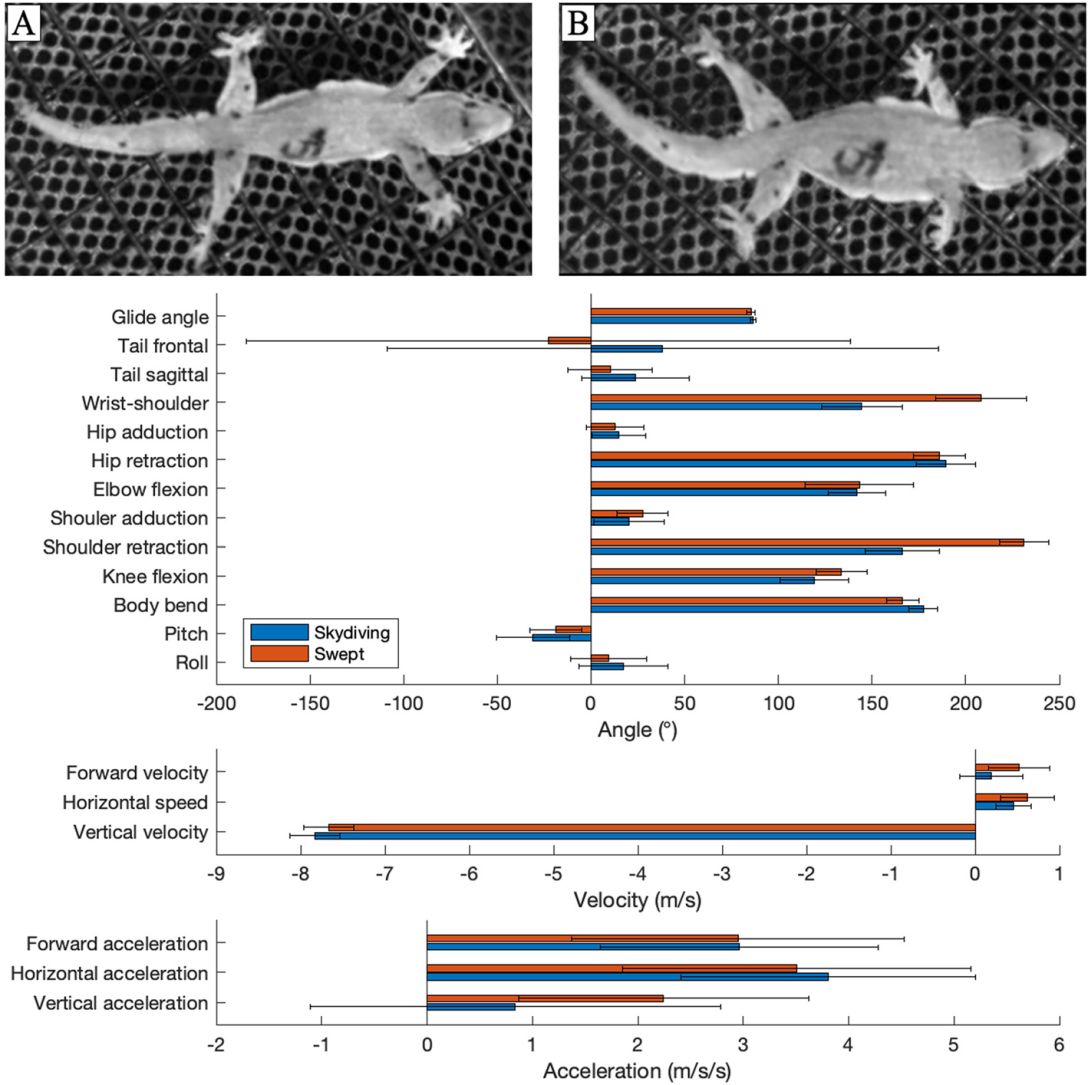
Skydiving and swept configuration postures and measurements of gliding geckos. **A** Photograph of a gecko gliding in the wind tunnel in skydiving posture (top view). Black marks on gecko represent the 14 tracked points (See Supplemental Materials) and the gecko’s identification number. **B** Photograph of a gecko gliding in the wind tunnel in swept configuration (top view). **C** Mean (± s.d.) values of body angles, velocities, and accelerations between the skydiving and swept configurations (*n* = 20)

Here, we characterize limb and body kinematics of gliding in an arboreal gecko (*Hemidactylus platyurus*) which glides using a stereotypical skydiving posture ([16–21] see Fig. 1). Via lateral extension of fore- and hindlimbs, these geckos maximize projected surface area and associated aerodynamic force production while gliding [4, 22]. Our primary hypothesis was that large-amplitude movements of the forelimbs while gliding in this posture can be used to control body dynamics and aerodynamic force production. Yet animal movements are complex, so we also hypothesize that body pitch and body bend will also correlate with aerodynamic force production and glide performance. We quantify biomechanical correlates of limb kinematics for gliding *H. platyurus* and describe consequences of forelimb reciprocation, demonstrating the aerodynamic utility of moving non-wing structures in a gliding tetrapod. Additionally, we characterize variation in forelimb motions given absence of information on this interesting behavior.

## Methods

### Study taxon and husbandry

*Hemidactylus platyurus* is an arboreal species with morphological adaptations that increase surface area (i.e., finger and toe webbing approximately one-third the length of the digits, ventrolateral skin folds on the trunk, and a dorsoventrally flattened tail; [23]). These adaptations likely act as basic airfoils [24] that enhance gliding performance [25] through decreased wing loading [26], although their aerodynamic function has been debated (see [20]). Individuals of *H. platyurus* have been observed falling with limbs outstretched [20] and documented to glide and have aerial control by using their tail as an inertial appendage to control pitch, roll, and yaw [17, 18, 27].

We used eight (5 male/3 female) adult flat-tailed house-geckos (*H. platyurus*) (mean body mass: 4.23 ± 0.92 g; mean SVL: 57.2 mm). All animals were acquired in June of 2018 from LLL Reptile. Geckos were housed in large mesh-walled tanks (width: 50.8 cm, length: 50.8 cm, height: 99 cm) within temperature-controlled rooms (24 °C) with a 12:12 h light:dark cycle. Each tank housed 4–5 individuals. All geckos were provided live crickets and water ad libitum. The ambient room temperature in which experiments were conducted was approximately 25 °C. All procedures were approved by the University of California, Berkeley Animal Care and Use Committee.

### Experimental procedures

We used the same equipment and similar procedures as we used in previously published studies [13, 28] (see Fig. 1 in [13] for illustration). In lieu of dropping the lizards from elevated heights (e.g., [19, 25]), we simulated gliding through use of a custom vertical wind tunnel (Wind Generator 01–10, Aerolab, Laurel, MD, USA; see Supplementary Materials). Since air flowing past a relatively stationary animal is aerodynamically equivalent to an animal moving at the same speed through still air, such a simulation provides equivalent ensuing forces in the air (e.g., [15]). This method both reduced risk of animal escape and enhanced our ability to film (see Movies S1-S2). Due to interindividual variation in lizard mass, we adjusted the nominal windspeed for equilibrium gliding to each individual.

We filmed each aerial event at 400 frames per second using three synchronized cameras (HiSpec 1 cameras, Fastec Imaging, San Diego, California, USA); one camera was positioned at dorsal (top; 960 × 990 pixels; 25 mm lens) perspective and two cameras were positioned at lateral perspectives, both of which were elevated ~ 20° above horizontal (front and side; 1024 × 992 and 832 × 872 pixels, respectively; 50 mm lenses) so as to increase visibility of anatomical landmarks. Given the filming speeds, additional tungsten lighting was needed from above and the sides. As in [13] and [28] we calibrated the cameras in pairs by recording and later digitizing checkerboard points via stereo image pairs [29]. We used footage of a polystyrene ball (diameter of 4 cm, mass of 0.034 g) falling vertically through the test arena, along with a horizontal arena edge, to define the global coordinate system.

**Fig. 2:**
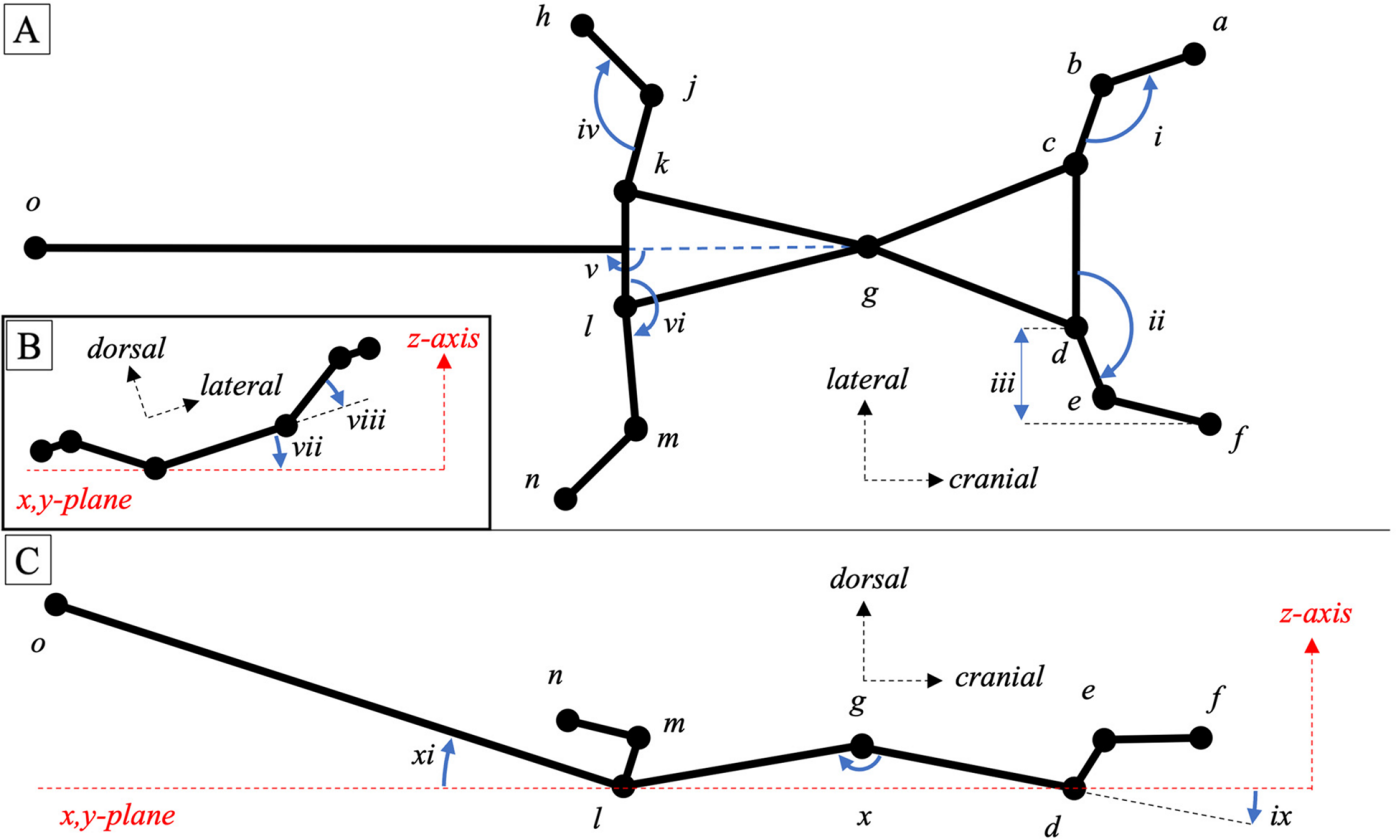
Postures and measurements of gliding geckos. **A** Idealized two-dimensional ball-and-stick representation of a gecko (top view). Black circles represent 14 of the 16 tracked points (the point between the eyes and the point midway along tail were not used): (a) left wrist; (b) left elbow; (c) left shoulder; (d) right shoulder; (e) right elbow; (f) right wrist; (g) midbody; (h) left ankle; (j) left knee; (k) left hip; (l) right hip; (m) right knee; (n) right ankle; and (o) tail tip. Sticks represent the relevant segments connecting points of interest. Black dotted arrows indicate anatomical directions. Blue arrows represent measured angles or distances: (i) elbow angle; (ii) shoulder retraction angle; (iii) wrist position; (iv) knee angle; (v) tail angle (frontal); (vi) hip retraction angle. **B** Idealized two-dimensional ball-and-stick representation of a gecko (cranial view). Blue arrows indicate (vii) roll and (viii) shoulder abduction angle. **C** Idealized two-dimensional ball-and-stick representation of a gecko (lateral view). Red dotted-lines represent x,y-plane and z-axis. Blue arrows indicate (ix) pitch; (x) body bend; and (xi) tail angle (sagittal)

Immediately prior to each gecko recording, we recorded the individual’s mass, and then applied non-toxic, high-contrast landmarks at relevant anatomical positions to the gecko’s skin (Figs. 1 and 2) with dark eyeliner (Megaliner, Ulta Beauty) or white paint pen (Garden Marker 450, Monami). We thus marked 14 total landmarks (but see Supplemental Materials): midway between the pectoral and pelvic girdle (or midbody), tail tip and on the wrist, elbow, shoulder, ankle, knee, and hip on both the left and right sides.

We recorded glides for each of the 8 individual geckos fifteen times in the wind tunnel (i.e., 120 videos total). The duration of each recording ranged from 1–10 s. To begin a recording, the gecko was released by hand into the airflow at a height of approximately 25 cm above the arena's floor. The recording was terminated within ~ 5 s of the start of a gliding event (i.e., traversing horizontally across any portion of the test section) or if the gecko halted its glide by either adhering to an arena wall or gripping the arena’s floor. The geckos did not always perform a gliding event or a limb reciprocation behavior of interest, so we only digitized a subset of all recorded sequences.

First, we identified those videos in which the gecko performed a gliding event. Among those videos, we removed any videos whereby the anatomical landmarks were not visible in at least 40% of frames or at the start and end of a gliding event in at least two cameras; a gliding event began when the gecko lost physical contact with the arena’s walls, floor, or with the researcher’s hand, and ended when the gecko regained contact with the arena. We then reexamined each of these gliding events and selected those recordings in which the geckos performed large and reciprocating forelimb motions relevant to our hypothesis. A total of 32 trials met these criteria from which we quantified limb and body motions. Among these 32 trials, points were visible in two cameras for an average of 95.6% frames.

### Kinematics measurements

For each of the 32 trials, we identified the frames in which the gliding event occurred, and tracked anatomical landmarks using DLTdv7 point-tracking software in MATLAB (The MathWorks; [30, 31]) in all three cameras. We used a checkerboard calibration routine in MATLAB to calibrate the cameras; the mean reprojection error across all calibrations was 0.818 ± 0.44 mm. We only used two-camera pairs (i.e., front and side, front and top, and side and top) to triangulate the 3D coordinates for analysis because the cameras differed in frame size and two cameras provided sufficient positional characterization of landmarks. For each trial, we selected the camera pair with the lowest amount of landmark occlusion and the smallest digital reprojection error. Using the ‘triangulate’ function in MATLAB, we reconstructed the three-dimensional point coordinates of the digitized landmarks for the corresponding camera pair. We smoothed positional data with a quintic spline smoothing algorithm [32], adjusting the tolerance value for each trial according to fit using the ‘splinetool’ function in MATLAB. We used this function to interpolate positional data for frames in which landmarks were occluded and used the first- and second- derivatives of each spline to approximate the instantaneous velocity and acceleration, respectively [32]. We calculated roll and pitch of the body, as well as (for both the left and right sides of the gecko) the angles of shoulder retraction, shoulder adduction, elbow flexion, hip retraction, hip adduction, knee flexion, and of the tail relative to both the sagittal and frontal plane (summarized here; see Supplementary Materials and Fig. 2 for details) in all 32 trials so as to quantify overall aerial performance.

To characterize body orientations and limb angles through time, we used vectors between relevant landmarks and two virtual planes: (1) the chest plane, which was bounded by the left shoulder, right shoulder, and midbody, and (2) the abdominal plane, which was bounded by the left hip, right hip, and midbody. To calculate body roll, we found the midpoint between the shoulders (i.e., midshoulder) and created a vector between it and the midbody point. We computationally aligned this vector with the global x-axis and measured the angle between the resulting chest plane and horizontal, such that a roll to the gecko’s right was represented by a negative angle, and a roll to the left was positive. Likewise, to calculate body pitch, we aligned the shoulders with the global y-axis and measured the angle between the chest plane and horizontal; positive pitch angle represents nose-up pitch, and negative pitch is a nose-down orientation. Note, however, that the mid-body point was slightly dorsal to the shoulder points, so when the chest plane was parallel to the horizon, the gecko would exhibit a slightly positive pitch (pitch up); alternatively, if the gecko itself were parallel to the horizon, body pitch would be slightly negative. This observation means that pitch values are skewed slightly negative, which introduces a small error in calculations for which the chest plane was used.

We measured body bend at the midbody point by identifying the midpoint between the hips (midhip), projecting it onto the chest plane, and measuring the angle bound by these three points (with its vertex at midbody). We added 180° to this angle such that body bend angles less than 180° represent a flexed vertebral column (i.e., with hips below the chest plane), and angles more than 180° represent vertebral extension (i.e., with hips above the chest plane). We considered the tail angle on the frontal plane as the clockwise rotation of the tail about the mid-hip point, whereby an angle of 180° represents the directly posterior position and angles < 180° and > 180° represent right and left displacement, respectively. To calculate the angle of the tail in the sagittal plane, we determined the angle between the tail and the abdominal plane, whereby negative and positive angles are below and above the abdominal plane, respectively.

To estimate shoulder adduction angle, we calculated the angle between the humerus and the chest plane. Negative and positive angles represent ventral and dorsal positions, respectively. We computed the shoulder retraction as the angle between the line connecting the shoulders and the humerus, as projected onto the chest plane. A positive shoulder retraction angle represents an anterior position, and a negative angle represents a posterior position. Similarly, we used the left hip, the right hip, and the midbody to define an abdominal plane which we coordinated with the knee and the hip axes to calculate hip adduction and retraction. To estimate elbow flexion angles, we calculated the angle between the humerus and the vector defined by the elbow and the wrist for the right and left forelimbs. Similarly, we estimated knee flexion angles using the femur and the vector defined by the knee and ankle. Since shoulder and elbow actuation can both change hand position, we also quantified the position of the wrist relative to the shoulder using the same method as with shoulder retraction, but using the wrist instead of the elbow.

For velocity estimates, we used the x-, y-, and z-components of the midbody because it was the landmark nearest to the center of body mass (i.e., approximately midway between the forelimbs and hindlimbs; see [33]). Vertical speed was taken to be the z-component of the body velocity. To calculate horizontal speed, we used the square root of the sum of the squares of the x- and y-components, which we then used to calculate forward speed, defined here as the horizontal speed in the direction of the gecko’s body axis such that a positive forward speed was in the gecko’s cranial direction. We estimated glide angle as the angle between the vertical speed and the horizontal speed, and considered forward speed, vertical speed, and glide angle to be the primary measures of glide performance. Similarly, we used the x-, y-, and z-components of midbody acceleration to calculate analogous acceleration values.

### Aerial postures and limb retraction

Since the kinematic focus of this study was on the novel behavior whereby geckos carried out symmetric and large-amplitude limb motions, we used Pearson correlation tests to detect bilaterally symmetric patterns in limb movements to decide which trials should be used for more detailed analyses, and to characterize gliding postures, detailed below as “skydiving” and “swept configuration” (see Fig. 1; Table 1). A limb motion was considered bilaterally symmetrical if the left and right sides showed a significant and positive correlation for that motion (e.g., shoulder retraction angle). Although we observed that the forelimb motions were far more dramatic than those of the hindlimbs, we nonetheless tested for bilateral symmetry between the left and right hip angles in each of the 32 trials. Whereas many of the trials demonstrated correlations between the left and right side for both the retraction and adduction angles, the signs of those movements were inconsistent and resulted in similar proportions of symmetrical and asymmetrical movements (Table 2). Since we were specifically interested in characterizing symmetric limb movements in this study, we did not further detail hindlimb kinematics.

**Table 1:** Posture values and comparison of skydiving and swept configuration postures

Variable	Skydiving Mean	Swept Mean	Test name	Test *p*-value
Roll (°)	17.2 (± 23.7)	9.4 (± 20.3)	t-test	0.109
Pitch (°)	−31.1 (± 19.5)	−18.6 (± 13.9)	t-test	**0.030**
Bend (°)	177.4 (± 7.8)	166.4 (± 8.5)	t-test	** < 0.001**
Shoulder retraction (°)	166.2 (± 19.9)	231.1 (± 13)	t-test	** < 0.001**
Shoulder adduction (°)	20.3 (± 18.6)	27.5 (± 13.7)	t-test	0.118
Elbow flexion (°)	142 (± 15.3)	143.3 (± 29.1)	t-test	0.793
Hip retraction (°)	189.5 (± 16)	186.2 (± 13.8)	t-test	0.204
Hip adduction (°)	15 (± 14.4)	12.9 (± 15.2)	t-test	0.520
Knee flexion (°)	119.3 (± 18.2)	133.7 (± 13.6)	t-test	** < 0.001**
Wrist shoulder adduction (°)	144.7 (± 21.4)	208.3 (± 24.2)	Wilcoxon	** < 0.001**
Tail sagittal (°)	23.8 (± 28.5)	10.2 (± 22.5)	t-test	0.074
Tail frontal (°)	182.3 (± 43.4)	175.3 (± 29)	t-test	0.480
Vertical velocity (m/s)	−7.8 (± 0.3)	−7.7 (± 0.3)	t-test	** < 0.001**
Horizontal speed (m/s)	0.5 (± 0.2)	0.6 (± 0.3)	t-test	** < 0.001**
Forward velocity (m/s)	0.2 (± 0.4)	0.5 (± 0.4)	t-test	** < 0.001**
Glide angle (°)	86.7 (± 1.5)	85.4 (± 2.3)	t-test	** < 0.001**
Vertical acceleration (m/s^2^)	0.8 (± 1.9)	2.2 (± 1.4)	t-test	** < 0.001**
Horizontal acceleration (m/s^2^)	3.8 (± 1.4)	3.5 (± 1.7)	t-test	0.301
Forward acceleration (m/s^2^)	3 (± 1.3)	3 (± 1.6)	t-test	0.971

Posture values represent mean (± s.d.) and *p*-values are from paired t-tests or Wilcoxon signed-rank tests comparing postures among the 20 trials selected for analyses. *P*-values indicating a statistically significant difference are in boldface

**Table 2:** Results from Pearson Correlation Tests between left and right shoulder retraction and between left and right hip retraction for all reconstructed trials

ID	Trial	Shoulder Retraction R^2^	Shoulder Retraction *p*-value	Shoulder Sweep Amplitude (°)	Hip Retraction R^2^	Hip Retraction *p*-value	Hip Adduction R^2^	Hip Adduction p-value
1	03A†	0.08	** < 0.001**	55.55	−0.06	**0.002**	0.00	0.855
1	03B†	0.13	** < 0.001**	56.69	0.17	** < 0.001**	0.57	** < 0.001**
1	07X	−0.03	0.107	-	0.00	0.746	0.01	0.462
1	11A†	0.81	** < 0.001**	72.49	−0.21	** < 0.001**	0.03	0.079
2	05A	0.00	0.643	-	−0.19	** < 0.001**	0.59	** < 0.001**
2	05B†	0.68	** < 0.001**	54.27	0.06	**0.009**	0.48	** < 0.001**
2	07A	0.11	**0.005**	-	0.50	** < 0.001**	0.93	** < 0.001**
2	07B†	0.89	** < 0.001**	72.28	0.27	** < 0.001**	0.30	** < 0.001**
2	09X	0.14	** < 0.001**	-	−0.09	** < 0.001**	0.20	** < 0.001**
3	09X†	0.40	** < 0.001**	45.37	0.16	** < 0.001**	0.01	0.261
3	11A†	0.93	** < 0.001**	93.27	−0.01	0.333	0.02	0.116
3	11B†	0.56	** < 0.001**	86.91	0.00	0.708	0.25	** < 0.001**
4	04X	−0.05	0.079	-	0.00	0.777	0.62	** < 0.001**
4	10X	−0.09	**0.004**	-	0.33	** < 0.001**	0.03	0.101
4	11X†	0.60	** < 0.001**	43.91	−0.97	** < 0.001**	0.99	** < 0.001**
5	03X†	0.31	** < 0.001**	41.81	0.08	**0.002**	0.15	** < 0.001**
5	06X†	0.42	** < 0.001**	47.02	0.16	** < 0.001**	0.12	** < 0.001**
5	08X†	0.61	** < 0.001**	99.58	0.04	**0.019**	0.56	** < 0.001**
5	10B†	0.59	** < 0.001**	96.29	−0.26	** < 0.001**	0.11	** < 0.001**
6	03X	−0.08	**0.012**	-	0.04	0.065	0.56	** < 0.001**
6	04A†	0.19	** < 0.001**	75.61	0.03	**0.019**	0.10	** < 0.001**
6	04B†	0.87	** < 0.001**	63.10	−0.04	**0.010**	0.15	** < 0.001**
6	05X	0.41	** < 0.001**	-	0.64	** < 0.001**	0.39	** < 0.001**
6	07X	−0.01	0.403	-	0.48	** < 0.001**	0.00	0.929
9	07A†	0.29	** < 0.001**	64.45	−0.03	0.050	0.73	** < 0.001**
9	07B†	0.96	** < 0.001**	54.35	−0.20	** < 0.001**	0.38	** < 0.001**
9	08X	−0.64	** < 0.001**	-	−0.42	** < 0.001**	0.46	** < 0.001**
9	10X	0.00	0.989	-	0.74	** < 0.001**	0.01	0.620
10	06X†	0.81	** < 0.001**	64.90	0.88	** < 0.001**	0.00	0.605
10	08X	0.54	** < 0.001**	-	0.10	0.087	0.10	0.091
10	09A†	0.54	** < 0.001**	56.88	−0.29	** < 0.001**	0.14	** < 0.001**
10	09B†	0.76	** < 0.001**	53.46	−0.04	0.087	0.87	** < 0.001**

The sign of the R^2^ value indicates negative and positive correlations. *P*-values indicating a statistically significant correlation are in boldface. The 20 trials with a positive and statistically significant correlation and through which the difference between the absolute maximum and absolute minimum shoulder retraction angle is greater than 45° are indicated with “†”; shoulder sweep amplitude is included for those trials

For the forelimbs, the change in shoulder retraction angle was noticeably more dramatic than that of the shoulder adduction angle (Movie S1-S2; Table 1). Thus, we only tested for bilateral symmetry between the left and right shoulder retraction angles (Table 2), opting to instead include shoulder adduction angle as a dependent variable in downstream analyses. Shoulder retraction angles were significantly and positively correlated (*p* > 0.004 in all cases), with correspondingly symmetrical movements, in only 24 of 32 trials. Of these, we used only those trials in which at least one of the shoulders moved through a retraction angle of at least 45º, thus eliminating all trials in which the shoulder retraction angles were either small or not positively correlated (i.e., not symmetrical) for detailed characterization of the forelimb retraction behavior. 20 trials were thus identified further kinematic analysis (Table 2). We used limbs from only one side per trial for analysis, selecting the side for which the forelimb was more representative of the retraction behavior (e.g., moved through a greater angle). In total, we analyzed 12 right forelimb sweeps and 8 left forelimb sweeps (Table 3).

**Table 3:** Information about the use of each trial selected for analyses

ID	Trial	Forelimb	Recovery Stroke	Multiple Retractions	Swept Oscillations
1	03A	Right	Present	-	Present
1	03B	Right	-	-	Present
1	11A	Left	-	-	Present
2	05B	Left	-	-	-
2	07B	Right	-	Present	Present
3	09X	Left	Present	Present	Present
3	11A	Right	-	-	Present
3	11B	Right	Present	-	-
4	11X	Left	Present	-	Present
5	03X	Right	Present	-	-
5	06X	Left	-	-	Present
5	08X	Right	-	Present	-
5	10B	Left	Present	Present	Present
6	04A	Left	Present	-	Present
6	04B	Left	Present	-	-
9	07A	Right	-	-	Present
9	07B	Right	-	-	-
10	06X	Right	-	-	Present
10	09A	Right	Present	-	Present
10	09B	Right	-	Present	Present

Forelimb used is included for all trials. Presence and absence is indicated for each trial of the recovery stroke, multiple shoulder retractions, and low-amplitude oscillation in the swept position behaviors

We considered the starting point for forelimb sweep to be either the local minimum immediately prior to large-amplitude shoulder retraction (Figure S1) or the first recorded frame. The end of forelimb sweep was defined as the first local maximum following shoulder retraction. We calculated the sweep amplitude as the difference between retraction angles at the start and at the end of this behavior. The half-period of the behavior was calculated as the difference in time between its start and end.

Qualitative observation suggested that large-amplitude shoulder retraction enabled transition between two primary gliding postures that we thereafter referred to as “skydiving” and “swept configuration”. The skydiving posture is comparable to that previously described in the literature [19, 21], with the body and tail flattened and the limbs laterally extended. Here, we distinguish the skydiving posture from the swept configuration primarily by the position of the forelimbs. Qualitatively, the forelimbs are held in a protracted (anterior) position in the sky-diving posture, whereas in the swept configuration, the forelimbs are retracted posteriorly. We used the single frame in which the local minimum marking the start of the shoulder sweep for the skydiving posture, and the local maximum for the swept configuration (Figure S1). At each of these timepoints, we characterized overall posture of the geckos by determining mean values of the pose variables among the trials (Table 1; Fig. 2). We then compared mean values for each of these variables using paired t-tests between skydiving and the swept configuration if the variable was normally distributed (determined by Lilliefors test; Table S1), or with a Wilcoxon signed-rank test if not (Table 1).

### Variation in forelimb behaviors

Behavioral variation in forelimb retraction was evident among trials (see Table 3). In nine of 20 trials, geckos performed a recovery stroke before the end of data collection, whereby the limbs exhibited substantial protraction towards the skydiving position (assessed visually, e.g., Figures S2-S3; Table 4). In all of these trials, the limbs returned to within 36% of each particular trial’s sweep amplitude relative to the initial retraction angle, as measured at a local minimum after the sweep or at the last frame of data. From these data, we determined the sweep duration. We characterized the posture at the end of the recovery stroke with the same measurements as were used for the skydiving and swept configuration postures. For these nine trials, we performed a repeated-measures ANOVA and Tukey–Kramer posthoc-analyses to compare the skydiving posture, swept posture, and “recovery” postures if the variable was normally distributed (determined by Lilliefors test; Table S1), or with a Friedman test and Dunn’s posthoc analyses if not (Table 4).

**Table 4:** Posture values for skydiving and swept configuration postures as well as those at the end of the recovery stroke

Variable	Skydiving mean	Swept mean	Recover mean	Test name	Test p-value	Posthoc Name	Sky-Swept *p*-Value	Sky-Recover *p*-Value	Swept-Recover *p*-Value
Roll (°)	25.00 ± (26.36)	15.08 ± (21.06)	15.45 ± (18.43)	Friedman	1	None	-	-	-
Pitch (°)	−41.72 ± (21.64)	−18.44 ± (17.86)	−23.42 ± (15.61)	ANOVA	**0.028**	Tukey	0.081	0.104	0.79
Bend (°)	178.52 ± (5.88)	168.48 ± (8.53)	173.02 ± (7.39)	ANOVA	**0.009**	Tukey	**0.017**	0.152	0.325
Shoulder retraction (°)	173.36 ± (17.95)	236.19 ± (14.33)	179.02 ± (21.34)	ANOVA	** < 0.001**	Tukey	** < 0.001**	0.682	** < 0.001**
Shoulder adduction (°)	15.05 ± (23.50)	27.33 ± (17.58)	27.93 ± (12.10)	Friedman	0.121	None	-	-	-
Elbow flexion (°)	142.75 ± (17.67)	133.92 ± (34.93)	140.61 ± (22.38)	ANOVA	0.472	None	-	-	-
Hip retraction (°)	190.73 ± (21.63)	181.47 ± (16.36)	183.95 ± (20.20)	ANOVA	0.39	None	-	-	-
Hip adduction (°)	17.93 ± (16.44)	16.56 ± (18.02)	14.84 ± (17.92)	ANOVA	0.923	None	-	-	-
Knee flexion (°)	118.68 ± (17.93)	137.93 ± (9.39)	130.14 ± (14.50)	ANOVA	**0.008**	Tukey	**0.006**	0.263	0.231
Wrist shoulder adduction (°)	153.77 ± (25.56)	205.18 ± (27.32)	153.40 ± (18.92)	ANOVA	** < 0.001**	Tukey	**0.005**	0.999	** < 0.001**
Tail sagittal (°)	16.34 ± (27.14)	10.46 ± (32.28)	30.95 ± (18.67)	ANOVA	0.159	None	-	-	-
Tail frontal (°)	177.89 ± (43.62)	168.58 ± (33.59)	156.48 ± (48.78)	Friedman	0.717	None	-	-	-
Vertical velocity (m/s)	−7.87 ± (0.25)	−7.73 ± (0.26)	−7.53 ± (0.39)	ANOVA	** < 0.001**	Tukey	0.061	**0.009**	**0.022**
Horizontal speed (m/s)	0.44 ± (0.26)	0.59 ± (0.30)	0.87 ± (0.45)	ANOVA	** < 0.001**	Tukey	**0.012**	**0.002**	**0.007**
Forward velocity (m/s)	0.22 ± (0.39)	0.52 ± (0.33)	0.72 ± (0.43)	ANOVA	** < 0.001**	Tukey	**0.041**	**0.015**	**0.011**
Glide angle (°)	86.75 ± (1.90)	85.64 ± (2.30)	83.35 ± (3.73)	ANOVA	** < 0.001**	Tukey	**0.01**	**0.004**	**0.011**
Vertical acceleration (m/s^2^)	0.91 ± (2.52)	2.35 ± (1.73)	1.07 ± (2.04)	ANOVA	0.08	None	-	-	-
Horizontal acceleration (m/s^2^)	4.13 ± (1.42)	3.55 ± (1.77)	2.96 ± (1.51)	Friedman	0.236	None	-	-	-
Forward acceleration (m/s^2^)	3.26 ± (1.49)	2.69 ± (1.76)	2.56 ± (1.52)	Friedman	0.368	None	-	-	-

Values represent mean (± s.d.). *P*-values derive either from repeated-measures ANOVAs and Tukey–Kramer posthoc-analyses, or from Friedman tests and Dunn’s posthoc-analyses comparing the postures among the 20 trials selected for analyses. P-values indicating a statistically significant difference are given in boldface

Occasionally, the gliding event was long enough to capture multiple shoulder retractions. Only two trials (gecko 3: trial 9 and Gecko 5: trial 10B) included two clear local maxima (e.g., Figure S1) indicative of the end of limb retraction. In addition to these trials, we analyzed three trials that captured a full sweep and also a sweep segment wherein the local maximum occurred prior to the onset of data collection, but for which the included data were greater than the local maximum used to define the sweep (e.g., Figure S2). Whereas this approach does not capture the entire behavior (and thus overestimates the effective reciprocation frequency), it does demonstrate the frequency at which the gecko could potentially sweep its arm from the maximum value to the minimum and then back to the maximum. The inverse of this period of sweeping motion was assumed to indicate such a potential reciprocation frequency.

In some trials, the gecko did not return their forelimbs to the skydiving position after the sweep either immediately (or at all), but instead oscillated the shoulders with low-amplitude changes in retraction angle (see Figure S1). We defined occurrence of this behavior as when trials showed a local minimum in shoulder retraction after the sweep, but either before a return to the skydiving position or with no such return. This behavior occurred for at least one cycle in 14 of 20 trials. As with forelimb sweeps, we recorded the amplitude and half period of these displacements, but we used the local maximum and minimum positions to indicate the start and end of the behavior, respectively.

### Statistical analysis

We performed Pearson correlation analyses between shoulder retraction angle and adduction angle. We also performed cross-correlation tests [34] in RStudio [35, 36] to identify relationships between the postural variables of shoulder retraction angle, pitch, and body bend (Table S2). For each of these postural variables, we performed cross-correlations with tail angle in the sagittal plane, forward velocity, vertical velocity, forward acceleration, and vertical acceleration (Tables S1-S2), so as to identify relationships between them while taking into account potential time lag between series (see [10]). Briefly, cross-correlation tests evaluate relationships between potentially lagged time series by staggering data incrementally across a variable number of time steps, and then generating correlation coefficients for each time step and corresponding lag (negative and positive lag values indicate that series 2 is shifted backwards or forwards in time, respectively, relative to series 1). However, because the relationships of interest were those of limb posture on glide performance, we only identified the positive lag for the closest and highest coefficient relative to zero lag. Finally, we performed a Pearson correlation test on the lagged dataset (see Fig. S4).

## Results

### Gliding dynamics and posture

In 32 reconstructed flight trials, geckos assumed a stereotypical skydiving posture with forelimbs projecting laterally, although this posture varied throughout the glide. Trials lasted between 75–590 ms, with an average of 272 ms. The mean shoulder retraction angle (all angles describing the dataset of 32 trials hereafter are averaged for both left and right limbs) throughout the glide across all trials was 196.4 ± 25.7°, indicating that the humeri were positioned slightly caudal to the shoulders with substantial anteroposterior displacement. The forelimbs were typically positioned dorsally relative to the shoulders at an adduction angle of 23.4 ± 12.7°. The femora were positioned slightly caudal to the hips with some anteroposterior displacement, given the hip retraction angle of 187.5 ± 12.4°. Similarly, the hip adduction angle was 10.2 ± 9.1°, with the femora being abducted dorsal to the hips. In the frontal plane, most geckos rotated their tails through high angles over the course of the trial; the maximum angle was 359.9° and the minimum angle was 10.4°. Mean tail angle (180.2 ± 14.7°) was almost directly posterior (i.e., 0°), indicating largely symmetric tail movements. Body pitch during glides varied substantially (−89.8° – 37.4°), with geckos on average decreasing pitch by −24.2° over the duration of the trial. Body roll also varied substantially throughout the trial, although relatively equal occurrences of leftward and rightward rolls resulted in a low average roll (mean of 7.4° ± 17.0°); maximum and minimum roll angles among all trials were 83.2° and −52.9°, respectively. Geckos nonetheless maintained a prone posture in the air, as neither absolute pitch nor absolute roll exceeded 90° for any trial.

Geckos descended at a mean vertical speed of −7.7 ± 0.3 m/s over the entire glide, whereas horizontal speeds averaged 0.6 ± 0.2 m/s, (maximum of −8.6 m/s), and the component of horizontal speed parallel to the longitudinal body axis (i.e., forward velocity) averaged 0.5 ± 0.3 m/s (maximum of 1.5 m/s). Mean glide angle across all trials averaged 85.3° ± 1.8°, with a minimum of 75.2°. Vertical accelerations averaged 1.2 ± 1.5 m/s^2^ (maximum of 6.0 m/s^2^) and horizontal accelerations averaged 3.6 ± 1.2 m/s^2^. The component of horizontal acceleration parallel to the longitudinal body axis (i.e., forward acceleration) averaged 2.89 ± 1.12 m/s^2^.

The variation in gliding posture is mainly a result of the geckos assuming two distinct aerial postures (Fig. 1; Table 1), here termed “skydiving” and “swept configuration” and illustrated using a subset of 20 trials in which the left and right forelimb retraction angles were positively correlated and were > 45° in amplitude (all angles describing the subset of 20 trials hereafter are for a single, designated limb; Tables 2 and 3).

Some kinematic features were not different between the skydiving and swept configuration postures and largely follow the overall trends described above (Table 1). Roll, shoulder adduction angle, elbow flexion, hip retraction, hip adduction, and tail angle in both planes were statistically non-different between postures. For both postures, the body was typically rolled slightly to the left although with notably high variation, the elbows were extended, the shoulders and hips were abducted above the body’s plane, the femora were positioned nearly parallel to the hips, and the tail was elevated above the abdominal plane and its lateral position varied substantially.

The two postures detailed here differed (Table 1) in pitch (*p* = 0.03), body bend (*p* < 0.001), shoulder retraction (*p* < 0.001), wrist position relative to the shoulder (*p* < 0.001), and knee flexion (*p* < 0.001). In both postures, the body is pitched downwards, but to a greater degree in the skydiving posture (−31.1 ± 19.5°) than in the swept configuration (−18.6 ± 13.9°). Likewise, the body was bent downwards (i.e., ventral flexion) in both postures, but the bend was less (i.e., flatter) in the skydiving posture (177.4 ± 7.8°) than in the swept configuration (166.4 ± 8.53°). In the skydiving posture, forelimbs were typically held in an anterior position (i.e., at a shoulder angle < 180°) relative to the shoulder joint (mean shoulder retraction angle of 166.2 ± 19.9°) and thus positioning the wrist and hands anterior to the shoulders (144.7 ± 21.4°), whereas in the swept configuration, the forelimbs (231 ± 13.0°) and hands (208.3 ± 24.2°) were held posteriorly. The knees were more flexed during the skydiving posture (119.3 ± 21.4°) and were straighter in the swept posture (133.7 ± 13.6°). Although tail positions in the two planes were statistically non-different, the p-value for the sagittal plane (*p* = 0.074) was marginally non-significant, giving some indication that the tail is held at a higher angle above the abdominal plane in the skydiving posture (23.8 ± 13.9°) than in the swept posture (10.2 ± 22.5°). In regard to the frontal plane, the tail seems to behave similarly between the skydiving posture (182.3 ± 43.4°) and the swept posture (175.3 ± 29.0°).

Accordingly, various aspects of glide performance also differed between postures. With a faster descent velocity (−7.8 ± 0.30 m/s; *p* < 0.001) and slower horizontal speed (0.46 ± 0.21 m/s; *p* < 0.001), the glide angle was greater in the skydiving posture (86.7 ± 1.5°; *p* < 0.001) than in the swept configuration (descent velocity = −7.7 ± 0.30 m/s; horizontal speed = 0.62 ± 0.32 m/s; glide angle = 85.4 ± 2.29°). There was also a greater forward velocity in the swept configuration (0.19 ± 0.38 m/s; *p* < 0.001) relative to the skydiving posture (0.52 ± 0.37 m/s). However, only vertical and not horizontal acceleration differed between the postures (*p* < 0.001), such that the value for the former parameter in the skydiving posture (0.84 ± 2.0 m/s^2^) was ~ 37% of that for the swept configuration (2.3 ± 1.4 m/s^2^).

### Forelimb kinematics

In 27 of 32 total trials (i.e., ~ 84%), motions of the left and right shoulders were significantly correlated (Table 2). Large (i.e., > 45°) symmetric sweeps of the forelimbs occurred in 20 of those trials (Table 2), which were selected for further detailed analysis. Among the 20 trials with symmetric and large-amplitude shoulder retractions (Table 2), the mean sweep amplitude was 65.0 ± 17.7°. Shoulder sweeps were initiated from the skydiving posture (at an average shoulder angle of 166.2 ± 19.9°) and ended in a swept configuration (mean shoulder angle of 231°); this motion, or the half period, was completed in 0.10 ± 0.05 s. Whereas the forelimbs moved in a stereotypic, symmetric pattern, hindlimb motions were inconsistent. Of 32 total trials, left and right hip retraction angles were significantly correlated in 25, but 11 (i.e., 44%) and 14 (i.e., 66%) were negatively and positively correlated, respectively (Table 2).

In a subset of the 20 trials with symmetric forelimb sweeps, geckos also performed either a recovery stroke or repetitive forelimb sweeps. The recovery stroke, during which the shoulder retraction angle returned to within 40% of its initial angle (Figure S1; Tables 3 and 4), occurred in nine trials (average 3.1 ± 35.2%). The average sweep duration from onset to end of the recovery stroke was 0.24 ± 0.07 s. At the end of the recovery stroke for these nine trials, the shoulder retraction (179 ± 21.34°) and wrist position relative to the shoulder (153.40 ± 18.92°) were statistically equivalent (*p* = 0.682 and 0.999, respectively) to that in the skydiving posture, but were significantly different from those in the swept configuration (*p* < 0.001 for both). The pitch (−23.42 ± 15.61°; *p* = 0.790), body bend angle (173.02 ± 7.39°; *p* = 0.325), and knee flexion angle (130.14 ± 14.50°; *p* = 0.231) did not differ between the skydiving and recovery configurations. The descent velocity (−7.53 ± 0.39 m/s; *p* = 0.022) and glide angle (83.35 ± 3.73°; *p* = 0.011) continued to decrease and horizontal speed (0.87 ± 0.45 m/s; *p* = 0.007) and forward velocity (0.72 ± 0.43 m/s; *p* = 0.011) continued to increase from the swept posture to the end of the recovery stroke. Although vertical acceleration appeared to decrease from the swept posture to the end of the recovery stroke (1.07 ± 2.04 m/s^2^), there was no significant difference among postures (*p* = 0.08). Horizontal acceleration, also showed no significant differences among postures (*p* = 0.368). Thus, whereas the forelimbs returned to their initial position, the posture assumed after the forelimb sweep is not equivalent to the skydiving posture.

Repetitive forelimb sweeps were identified in five trials (e.g., Figure S1; Table 3). In four of these trials, the recording duration was less than 0.4 s and included either two beats, or one full and one partial beat, corresponding to an effective mean reciprocation frequency of 5.6 ± 2.3 Hz). In 14 trials, forelimbs were oscillated at low amplitude (i.e., < 45, with mean change in shoulder retraction angle of 9.4 ± 7.9°) while nominally held in the swept configuration (Table 3; Movie S3-S4). The average half period for these oscillations was 0.039 ± 0.029 s. In six of the 14 trials with such oscillations, a recovery stroke also occurred after either one (four trials) or two (two trials) oscillations. In the remaining eight trials, recording was terminated while the forelimbs were in the swept configuration after either one oscillation (six trials) or two oscillations (two trials).

### Shoulder and body kinematics

**Fig. 3:**
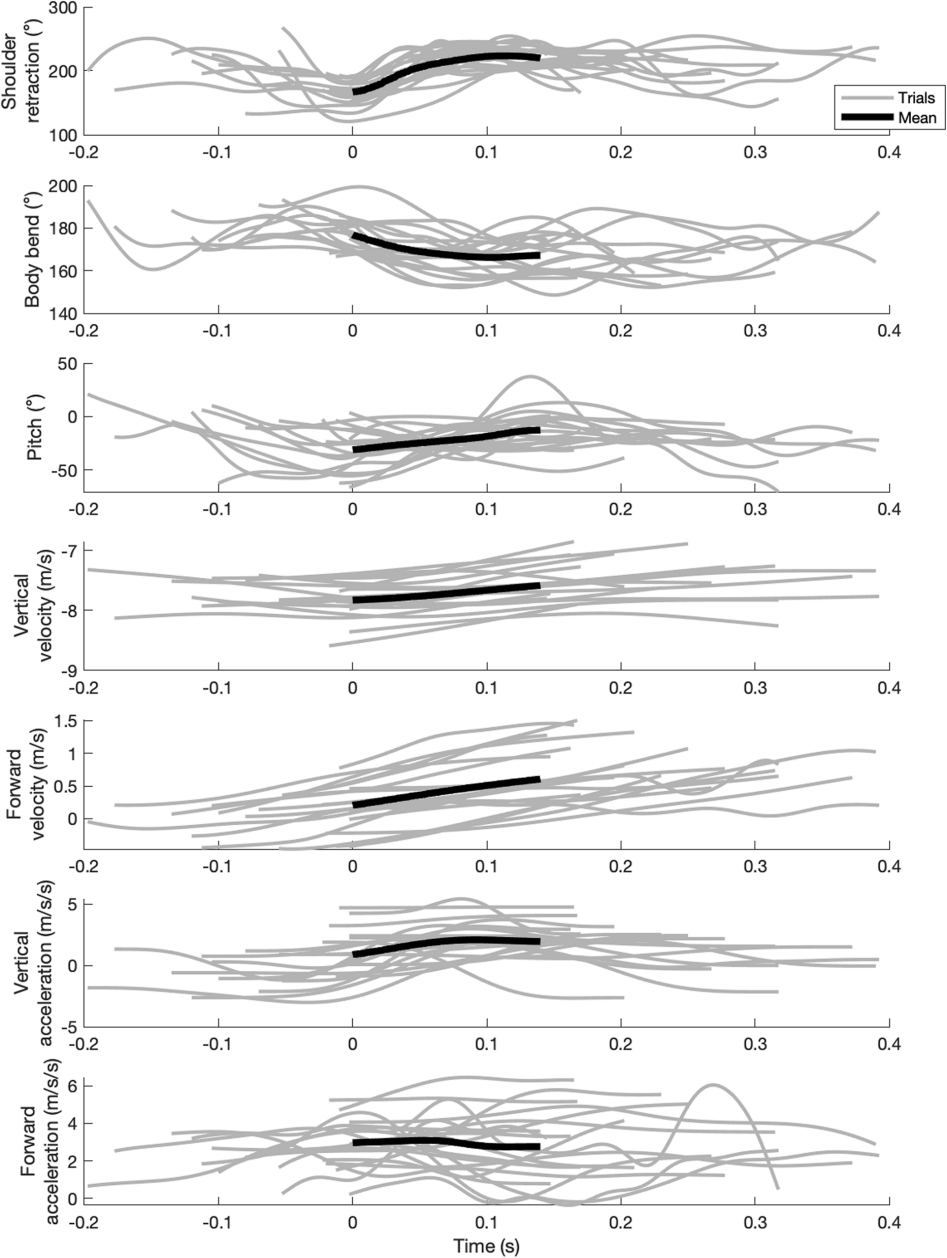
Shoulder retraction angle, body bend, pitch, velocities, and accelerations are correlated. Specifically, shoulder retraction showed positive relationships with forward velocity and vertical acceleration and a negative relationship with body bend; body bend showed negative relationships with forward velocity, vertical velocity, and vertical acceleration; pitch showed a positive relationship with vertical acceleration and a negative relationship with forward acceleration. Mean values are included only for times during which all trials (*n* = 20) yielded corresponding data (i.e., from time = 0 s to time = 0.14 s). Data for all trials were temporally synchronized such that the forelimb sweep initiates (i.e., skydiving posture terminates) at time = 0 s (see Tables S1-S2 for correlation data). At time = 0, geckos in each trial are in the skydiving posture, and then attain a swept posture within ~ 0.10 s on average

The results from the cross-correlation analyses for each trial are presented in Tables S2-S4 and are summarized in Table 5. In 19 of the 20 trials for which geckos performed large-amplitude, symmetric forelimb movements, shoulder retraction and shoulder adduction angles were significantly correlated (*p* ≤ 0.015 and R^2^ ≥ 0.30 for all significant trials; mean lag of 0.018 ± 0.034 s; see Table S2), though the only remaining trial was marginally non-significant (*p* = 0.054, R^2^ = 0.15, lag = 0.01 s). However, only 13 trials (including that which was marginally non-significant) demonstrated a positive correlation whereby the forelimbs moved from a position that was both anterior (i.e., protracted) and ventral (i.e., adducted) relative to the shoulders to a position that was both posterior (i.e., retracted) and dorsal (i.e., abducted). In 19 trials, shoulder retraction angle was significantly correlated with pitch (*p* ≤ 0.038; R^2^ ≥ 0.19, for all significant trials; mean lag of 0.026 ± 0.038 s; Table S2), although this relationship was negative (such that shoulder retraction coincides with or precedes pitch down) for only eight trials (Table 5). Similarly, shoulder retraction angle and body bend were significantly correlated in 19 trials (*p* ≤ 0.001 and R^2^ ≥ 0.43 for all significant trials; mean lag of 0.026 ± 0.031 s; Table S2). However, this relationship was negative in 18 trials (i.e., 95%; Table 5), indicating that as the shoulders retracted, the spine flexed ventrally and typically before the shoulders assumed a swept configuration (e.g., Fig. 3). The tail angle in the sagittal plane was correlated with shoulder retraction in 18 trials (*p* ≤ 0.002 and R^2^ ≥ 0.23, for all significant trials; mean lag of 0.019 ± 0.021 s; Table S4), with 14 of those correlations (i.e., 78%) showing a negative correlation whereby the tail moved more ventrally as the shoulders moved posteriorly.

**Table 5:** Summary of the cross-correlation analyses

			Significant and Positive				Significant and Negative			
Variable1	Variable2	Count Significant	Count	Mean Lag	Max. *p*-value	Min. R2	Count	Mean Lag	Max. *p*-value	Min. R2
Bend	Forward Acceleration	19	7 (36.8%)	0.000	< 0.001	0.39	12 (63.2%)	0.028	< 0.001	−0.53
Bend	Forward Velocity	19	5 (26.3%)	0.0205	0.019	0.23	**14 (73.7%)**	0.0036	0.007	−0.22
Bend	Pitch	19	10 (52.6%)	0.050	< 0.001	0.64	9 (47.4%)	0.021	< 0.001	−0.60
Bend	Vertical Acceleration	20	3 (15.0%)	0.0442	< 0.001	0.48	**17 (85.0%)**	0.0100	< 0.001	−0.50
Bend	Vertical Velocity	20	6 (30.0%)	0.0592	< 0.001	0.66	**14 (70.0%)**	0.0020	0.016	−0.21
Pitch	Forward Acceleration	20	5 (25.0%)	0.0555	< 0.001	0.89	**15 (75.0%)**	0.0128	0.008	−0.22
Pitch	Forward Velocity	20	13 (65.0%)	0.036	< 0.001	0.54	7 (35.0%)	0.010	< 0.001	−0.38
Pitch	Vertical Acceleration	20	**15 (75.0%)**	0.0192	0.0110	0.33	5 (25.0%)	0.0255	< 0.001	−0.49
Pitch	Vertical Velocity	20	11 (55.0%)	0.010	< 0.001	0.54	9 (45.0%)	0.053	0.006	−0.22
Shoulder Retraction	Bend	19	1 (5.3%)	0.0475	< 0.001	0.80	**18 (94.7%)**	0.0247	< 0.001	−0.43
Shoulder Retraction	Forward Acceleration	20	12 (60.0%)	0.050	0.0488	0.16	8 (40.0%)	0.026	< 0.001	−0.33
Shoulder Retraction	Forward Velocity	19	**15 (78.9%)**	0.0097	< 0.001	0.29	4 (21.1%)	0.0244	0.008	−0.20
Shoulder Retraction	Pitch	18	10 (55.6%)	0.012	< 0.001	0.55	8 (44.4%)	0.044	0.038	−0.19
Shoulder Retraction	Vertical Acceleration	19	**15 (78.9%)**	0.0220	0.010	0.18	4 (21.1%)	0.0506	< 0.001	−0.68
Shoulder Retraction	Vertical Velocity	20	13 (65.0%)	0.008	0.0022	0.26	7 (35.0%)	0.071	< 0.001	−0.40

All significant correlations that shared a sign in at least 67% of relationships are indicated in boldface

In all 20 trials, shoulder retraction and forward velocity were significantly correlated (*p* ≤ 0.001 and R^2^ ≥ 0.03 for all significant trials; mean lag of 0.012 ± 0.022 s; Table S2). 15 of these trials displayed a positive correlation whereby increased forward velocity accompanied shoulder retraction (e.g., Fig. 3; Table 5). Shoulder retraction angle was also significantly correlated with vertical velocity in all 20 trials (*p* ≤ 0.001 and R^2^ ≥ 0.26 for all significant trials; mean lag of 0.03 ± 0.043 s; Table S2), but correlation was only positive in 13 of them (Table 5). Shoulder retraction angle and forward acceleration were significantly correlated (*p* ≤ 0.001 and R^2^ ≥ 0.16 for all significant trials; mean lag of 0.04 ± 0.04 s; Table S2; Fig. 3) in all 20 trials; this relationship was positive for only 12 of the 20 trials (Table 5). Shoulder retraction angle was also significantly correlated with vertical acceleration in 19 trials (*p* ≤ 0.001 and R^2^ ≥ 0.18 for all significant trials; mean lag of 0.028 ± 0.37 s; Table S2), 15 of which were positive correlations whereby retracting the shoulders was followed by an increase in upwards force (Table 5).

Whereas all dependent variables often correlated with body bend, only forward velocity, vertical velocity, and vertical acceleration did so with inconsistent sign. Pitch was correlated with body bend in 19 of the trials (*p* ≤ 0.001 and R^2^ ≥ 0.60 for all significant trials; mean lag of 0.035 ± 0.041 s; Table 5; Table S2), but with 10 positive and nine negative the sign of those relationships was inconsistent. Body bend was significantly correlated with forward velocity in 19 trials (*p* ≤ 0.007 and R^2^ ≥ 0.22 for all significant trials; mean lag of 0.008 ± 0.016 s Table S3), and with a negative correlation in 15 (75%) of these. After the body flexed ventrally, horizontal speed increased with an average lag of 0.008 s. Vertical velocity also correlated with bend in all trials (*p* ≤ 0.016 and R^2^ ≥ 0.21 for all significant trials; mean lag of 0.019 ± 0.033 s; Table 5; Tables S1-S2), with 70% of those being negative. Geckos thus tended to descend faster when the spine was extended. Bend and forward acceleration were also significantly correlated in 19 trials (*p* < 0.001 and R^2^ ≥ 0.39 for all significant trials; Table S3), 12 of which showed a negative correlation indicating that spinal flexion yielded an increase in forward acceleration. However, bend and vertical acceleration were significantly correlated in all trials (*p* ≤ 0.001 and R^2^ ≥ 0.48 for all significant trials; Table 5; Table S3); the correlation was negative in 17 of these, suggesting that spinal extension reduced vertical acceleration (with a lag of approximately 0.01 s; Table S3). Bend was correlated with tail angle in the sagittal plane in 19 trials (*p* ≤ 0.030 and R^2^ ≥ 0.17 for all significant trials; mean lag of 0.019 ± 0.023 s; Table S4). Among those correlations, 15 (i.e., 79%) were positive, whereby the tail is depressed as the body bends downwards.

Body pitch was significantly correlated with all dependent variables (except tail angle in the sagittal plane) in all trials, but the sign was only consistent in forward and vertical acceleration. Body pitch was positively correlated with forward velocity in 13 gliding trials and negatively correlated in seven trials (*p* ≤ 0.002 and R^2^ ≥ 0.38 for all significant trials; mean lag of 0.027 ± 0.035 s; Table S3). With vertical velocity (*p* ≤ 0.006 and R^2^ ≥ 0.22 for all significant trials; mean lag of 0.030 ± 0.042 s; Table S3), pitch had only 11 (i.e., 55%) positive and nine negative correlations (Table 5). Pitch demonstrated consistent negative correlations with forward acceleration (75% of the 20 significant correlations; *p* ≤ 0.008 and R^2^ ≥ 0.22 for all significant trials; mean lag of 0.024 ± 0.035) and consistent positive correlations with vertical acceleration (75% of the 20 significant correlations; *p* ≤ 0.011 and R^2^ ≥ 0.33 for all significant trials; mean lag of 0.0209 ± 0.031 s; Table 5; Table S3). Pitch correlated significantly with tail angle in the sagittal plane in 18 trials (*p* ≤ 0.007 and R^2^ ≥ 0.22 for all significant trials; mean lag of 0.017 ± 0.031 s; Table S4). However, 50% of these correlations were positive.

In short, the body bends downwards and the tail becomes more aligned with the body when the forelimbs retract posteriorly. Forward velocity and upward acceleration correlate positively with both of these actions, as well as with upwards pitch. Whereas vertical velocity increases only with downward bend, forward acceleration correlates only with downward pitch.

## Discussion

This study demonstrates correlations between various limb and body kinematics of gliding geckos, and particularly among shoulder retraction, body bending, and forward speed. Whereas geckos primarily used a stereotypical skydiving posture to effect upright and stable gliding, they also exhibited substantial postural variation and carried out stereotypical reciprocating motions with the forelimbs in the anterior and posterior directions. As shoulder retraction angle increased during forelimb motion, body bending decreased and forward speed increased, supporting the hypothesis that these forelimb motions correlate with both body postural change and thrust generation. These kinematic changes also corresponded to an increase in upward acceleration of the body.

**Fig. 4:**
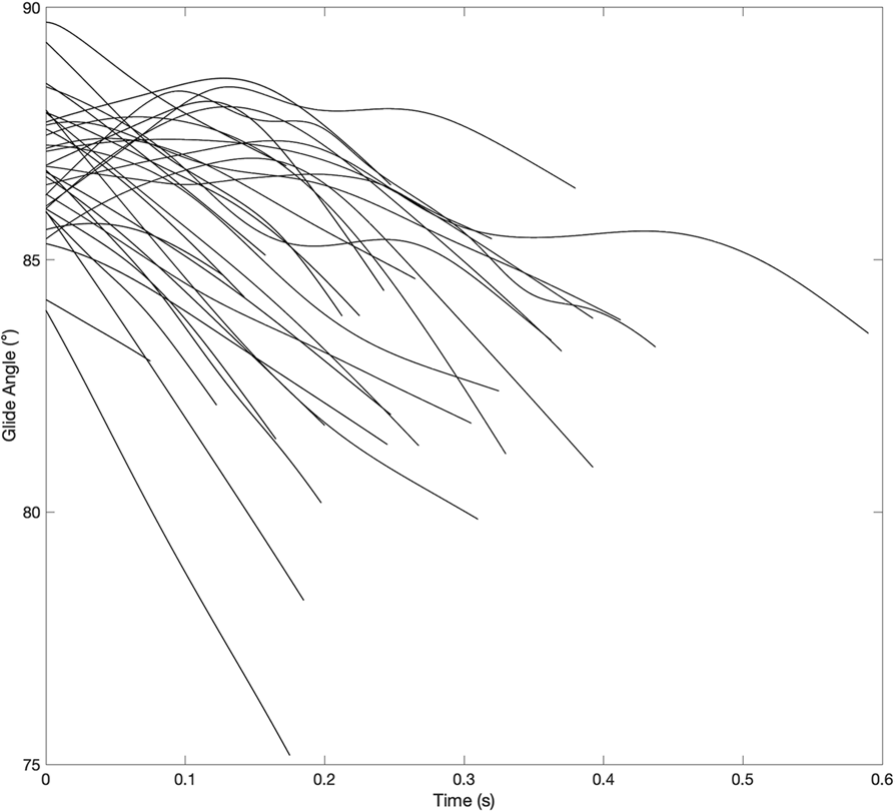
Glide angle of geckos decreased throughout the glide. Lines illustrate profiles of instantaneous glide angles during each trial

Here, geckos descended at steep although shallowing angles (~ 85º; Fig. 4). The maximum glide angle recorded here (75.2°) was less than the mean glide angle documented for *H. platyurus* in the field (~ 53º; [27]). Geckos typically accelerated through the glide and were thus not in force equilibrium (Figs. 2 and 3), consistent with the shallowing phase of an initial glide trajectory [37]. In all 32 trials, glide angles continuously decreased through time (by ~ 4° over ~ 270 ms). Glide angles described here are much steeper than those documented for taxa with prominent patagial membranes (e.g., [10, 14, 25, 37–39]), but are similar to those of taxa lacking specialized aerodynamic structures [13, 19, 28, 40]. We expect that glide angles would continue to decrease if the trajectories had not been halted by the arena walls. We thus acknowledge that the constraints of the experimental setup limited glide duration and horizontal translation, and we therefore only captured a relatively small component of the gliding behavior. Nonetheless, we captured sufficient glide durations so as to identify characteristic postures and movements, and the horizontal body accelerations reported here are consistent with aerodynamic lift production along with positional control derived from forelimb motions.

In particular, the swept forelimb configuration with retracted shoulders, a relatively flexed spine, and straightened knees (as distinct from the skydiving posture) was correlated with forelimb retraction and body bend (Fig. 3). Each limb can be viewed conceptually as a dorsoventrally compressed elliptic cylinder with a distal dorsoventrally compressed spheroid; the postures in which they are held can indeed have dramatic consequences for performance [14]. Posterior motions of the forelimbs would similarly alter the body’s center of aerodynamic pressure relative to the center of mass. Thus, with forelimbs held posteriorly, the center of aerodynamic pressure would move slightly backward and require a compensatory downward pitching torque [41]. Depressing the tail from a high angle in the sagittal plane closer to the body plane would similarly move the center of pressure posteriorly and would perhaps increase the surface area oriented towards oncoming airflow and upwards force as a consequence. Surprisingly, the body pitched relatively less downwards in the swept posture relative to the skydiving posture (Table 1). Pitching torques may have also been influenced by simultaneous body bending, with spinal flexion yielding a downward and backward motion of the posterior trunk (and thus center of body mass) in the global coordinate system. In turn, the center of body mass also moved posteriorly. Additionally, the knees extended further in the swept posture, which, in combination with the consistent hip adduction angle, would move the feet laterally and anteriorly. This motion may mitigate the downward pitch resulting from the posterior displacement of the forelimbs, and would maintain roll stability.

Abduction angle of the forelimbs was unchanged during posterior displacement (Table 1). Sustained shoulder abduction would elevate the center of aerodynamic pressure relative to the center of mass and would enhance stability; consistently positive adduction angles of the femora (Table 1) suggest a similar outcome for the hindlimbs. In gliding frogs, relative stasis of the hindlimbs is consistent with passive aerodynamic stability during stereotypical and stable skydiving [15, 19].

The posterior limb displacement and body bending during glides increased horizontal, forward, and vertical velocities and vertical accelerations such that the glide angle decreased (Table 1). Given its large surface area relative to the limbs and tail, the body is likely the primary lift generator in *H. platyurus*, and its ventral flexion in the swept configuration increased camber and presumably the lift:drag ratio. Additionally, although tail elevation in the sagittal plane was not statistically different between the postures, its pattern of being less elevated (i.e., in-line with the abdominal plane) in the swept configuration would increase the surface area facing oncoming airflow and enhance lift production. The dorsoventrally flattened forelimbs may also produce small useful aerodynamic forces. Given the combination of pitch and shoulder adduction angles in the skydiving posture, the forelimbs were approximately horizontal, and resultant aerodynamic force produced in this configuration would point upwards, medially, and with possibly a small anterior component. By contrast, resultant aerodynamic force with the forelimbs in the swept configuration would be directed upwards and medially, but with large anterior component that would accelerate the body forward. Furthermore, we observed that when in the skydiving posture, the hands would appear relatively level (i.e., parallel with the horizontal plane), whereas when in the swept configuration, the forelimbs would twist so that the hands would pitch downwards. Such a posture would direct forces produced by the hand anteriorly and would presumably contribute to forward acceleration; the effect of hand orientation on aerodynamic performance is a fruitful avenue for future study. Although the change in glide angle observed between the skydiving and swept postures is relatively small (~ 1.3°), even a small change in glide angle can be highly beneficial over biologically relevant scales by increasing access to favorable landing sites (e.g., [13]). Furthermore, by defining the swept configuration as the local maximum following retraction, this posture comparison only captures the very early effects of this postural change and does not account for any lag in aerodynamic force production; it is unlikely that changes in velocities or velocity-based performance metrics such as glide angle would be fully realized at this time point. Instead, the average 4° increase in glide angle over the glides’ duration is perhaps more indicative of the net consequences of this change in posture (Fig. 4).

The forelimb sweep behavior enabled transition between the skydiving and swept configurations and is highly associated with body bending, but not with pitch. Of course, gecko bodies are highly dimensional, dynamic, and unconstrained in the skydiving and swept postures; the ways in which they move their bodies are continuous and can have major consequences for gliding performance. Forward velocity often (i.e., ≥ 70% of the time) increased after posterior forelimb displacement and downward body bend, and vertical velocity often increased after downward body bend with minimal lag (i.e., < 10 ms; Table 5; Fig. 3). Forward speed increased faster than did vertical speed, or rather forward acceleration was generally higher than vertical acceleration, so glide angle consistently decreased over the glide duration (Figs. 2–3). These postural changes influence vertical accelerations, and pitch seems to alter both vertical and forward speeds as well, although we interpret accelerational correlations with caution given reconstruction noise in these data. Intriguingly, whereas neither shoulder retraction nor body bend were consistently correlated with forward acceleration, an upwards pitch was often followed by a decrease in forward acceleration and an increase in vertical acceleration. This pattern is consistent both with aerodynamic theory in that an upwards pitch increases the angle of attack which redirects lift force such that the forward component (i.e., thrust) decreases and the upwards component increases. Empirical studies on other gliding animals have similarly shown how changes in pitch or angle of attack redirect the aerodynamic force orientation (e.g., [11, 38, 42]).

After forelimb retraction, geckos returned the forelimbs to the skydiving posture position in 45% of trials, and occasionally performed multiple retractions (i.e., 25% of trials). Additional retractions may have occurred after the recovery stroke, but were not necessarily recorded in our experimental trials. Nonetheless, forelimb sweeps can be repeated multiple times during a glide. However, subsequent forelimb retractions may initiate from a posture that is not equivalent to the characterized skydiving posture (Table 4) because other postural measurements (such as pitch, body bend, and knee flexion) remain similar to those in the swept configuration, though all of these features show a trend of returning to their characteristic positions in the skydiving posture. Notably, the vertical velocity increased (i.e., descent slowed), forward velocity increased, and glide angle decreased starting from values in the skydiving posture, within the swept posture, and again to the end of the recovery stroke, suggesting that perhaps the persistent pitch body bend angle continued to facilitate forward and vertical force production, respectively, and thus contributed to the decreases in glide angle. The pattern for vertical acceleration is particularly intriguing when considering body bend and shoulder retraction. Whereas all three of these were significantly different between the skydiving posture and the swept configuration, the body bend angle and vertical acceleration when the forelimbs complete the recovery stroke did not differ from either the skydiving or the swept postures, although the vertical acceleration (1.07 m/s^2^) nearly returned to its initial skydiving value (0.91 m/s^2^), thus decreasing from the initial value in the swept posture (2.35 m/s^2^). This pattern more closely follows that of forelimb retraction than that of the body bend, further illustrating the link between these variables.

Whereas a single shoulder retraction can alter vertical and horizontal force production, more fine-scale control during a glide may be enabled by sustained reciprocations, as may low-amplitude oscillations of the forelimbs (~ 9°) in the swept configuration. Our criteria for trial selection, and an assumed threshold of 45º to delineate forelimb retraction, limit these findings to the relatively large-amplitude sweeps and their correlates with body posture, velocity, and acceleration. Nonetheless, forelimbs can be highly active in gliding *H. platyurus* and likely associate with control of body orientation via bilaterally symmetric force and torque generation.

The forelimb reciprocation motions documented here differ substantially in terms of frequency and amplitude relative to running kinematics of squamates [43, 44]. The anteroposterior angle through which the forelimb moves during gliding (~ 65º) is similar to that in running geckos (e.g., *Chondrodactylus bibronii*; [45]. In our study, the combined half period of the sweep (mean of ~ 0.1 s) and the duration of recovery stroke (mean of ~ 0.2 s, but much shorter durations were also observed, e.g., Figure S1) suggest that geckos could feasibly reciprocate their forelimbs between these postures at a frequency of ~ 3–5 Hz. Repeated forelimb sweeps at these frequencies occur well below running frequencies achieved by *Hemidactylus* spp. geckos (~ 9–15 Hz; [43]. Only the high-frequency (13 Hz) forelimb oscillations of swept configuration approach these stride frequencies, albeit at a much lower amplitude (~ 9°). Perhaps the starkest difference between the kinematics of the aerial forelimb reciprocation and running is that the reciprocation behavior uses bilaterally symmetric shoulder retractions, whereas running employs bilaterally asymmetric limb movements. Whereas the hindlimbs were somewhat active while gliding (Table 1), their motions were at times bilaterally asymmetric and with variable phase relationships to the forelimbs (Table 2). We suspect that bilaterally asymmetric fore- and hindlimb motions are related to steering (i.e., yaw control), and we plan to investigate this further in future studies.

These findings for gliding geckos have implications for our understanding of the biomechanical origins of flapping flight in vertebrates. The frequencies of forelimb retraction and recovery (4–5 Hz) are similar to the minimum frequency estimated to produce a jet wake with rudimentary wings while gliding [6], and are comparable to those postulated for paravian protofliers (i.e., 2–6 Hz; [1, 6]). However, sweep amplitudes characterized here were smaller than those postulated for avian protofliers [6], and were restricted to displacement within the frontal plane. Nevertheless, thrust generation via vortex wake production does not require substantial wing displacement, and would facilitate further elaboration of flapping flight [1, 2, 6, 46]. Furthermore, with gravitational potential energy being the primary energy behind aerodynamic forces, lift production would incur little metabolic cost to the animal [47]. Aerodynamic properties of the elongated and feathered forelimbs of avian precursors [48–55] would also be superior to those of the lizards studied here.

In extant taxa, equilibrium gliding postures generally are characterized by bilaterally symmetric deployment of wings, appendages, and patagia [16, 17, 19, 21]. In addition to symmetric postures, we characterized symmetric forelimb movements that a gliding animal can evidently use to modulate aerodynamic forces and potentially enhance trajectory control and as well as selection of landing location. The transition from the bilaterally asymmetric limb motions of cursorial locomotion to the symmetric flapping stroke remains unresolved, yet is fundamental to the origin of powered flight [6]. In addition to the symmetry inherent to the stable skydiving posture that is ubiquitous among gliding taxa, the symmetric forelimb movements described in this study suggest a plausible pathway by which the flapping stroke evolved through progressive increases in stroke amplitude and consequent improvements in producing controlling aerodynamic forces [1, 6]. As proposed for the origins of avian flight [1, 4, 6], symmetric forelimb reciprocation as described here would provide progressive and beneficial augmentation in flapping kinematics, with consequent enhancement of forward thrust over evolutionary time.

## Conclusions

Bilaterally symmetric postural adjustments of the body and forelimbs in geckos result in modulation of body dynamics and associated trajectories. Aerodynamic control in this case, including intentional changes in speed and body orientation, would likely provide selective advantages for any taxonomic group that becomes airborne. Thus, analogous motions (including symmetric forelimb reciprocation) would provide a plausible evolutionary pathway for volant vertebrates to effect a transition from the asymmetric limb motions of running to the symmetric flapping stroke in powered flight.

## Supplementary Information

Supplementary Material 1.

Supplementary Material 2.

Supplementary Material 3.

Supplementary Material 4: Movie S1. A glide by gecko 3 viewed from the top camera. The video begins with the gecko parachuting while in the skydiving posture with the hindlimbs held laterally and the forelimbs protracted slightly; the lizard is facing the side camera. The gecko strongly retracts its forelimbs and assumes the “swept configuration” and subsequently glides towards the side camera. As it glides, the gecko performs low-amplitude oscillations in the shoulder retraction angle while in the “swept configuration”. The gecko collides with the wall nearest the side camera. This video demonstrates the symmetrical, forelimb sweep behavior and the low-amplitude oscillations while in the swept configuration.

Supplementary Material 5: Movie S2. A glide by gecko 5 viewed from the top camera. The video begins with the gecko parachuting while facing the corner between the wall nearest the front camera and the wall opposite the side camera. The gecko had just begun to perform the shoulder retraction behavior and quickly enters the swept configuration. The gecko then begins to move in the forward direction and immediately protracts its shoulders and enters the skydiving posture. The gecko engages in anticlockwise tail rotations and turns to its left and faces the wall nearest the front camera but at an angle slightly towards the side camera. As it turns, it performs a second shoulder retraction event and subsequently increases speed in the forward direction. It partially protracts its limbs to ~180º and maintains this position until it collides with the wall. This trial demonstrates a full retraction, recovery stroke, and low-amplitude oscillations while in the swept configuration.

Supplementary Material 6: Movie S3. A glide by gecko 1 viewed from the top camera. The video begins with the gecko moving slightly backwards away from the wall nearest the front camera. The gecko is facing towards the front camera and is in the skydiving posture. The gecko then retracts its forelimbs and assumes the swept configuration. At the initiation of this behavior, the backslide slows down and the gecko gradually starts to move in the forward direction. While in the swept configuration, the gecko’s forelimbs oscillate with low-amplitude and the gecko accelerates in the forward direction until it collides with the arena walls.

Supplementary Material 7: Movie S4. A glide by gecko 4 viewed from the top camera. The video begins with the gecko adjacent to the wall farthest from the side camera and oriented away from the front camera, but with its head tilted towards the side camera. The gecko is moving in the forward direction and is engaged in a yawing turn towards the side camera with its forelimbs in the skydiving position. The gecko immediately retracts its forelimbs and assumes the swept configuration posture. It accelerates towards the side camera and its body aligns with the direction in which the head was facing initially, thus completing the yaw turn. The gecko continues to speed up in the cranial direction while engaging in low-amplitude oscillations in the swept configuration. The gecko completes a recovery stroke just before its left limbs make contact with the wall farthest from the front camera. This trial demonstrates a full retraction, recovery stroke, and low-amplitude oscillations while in the swept configuration.

Supplementary Material 8: Movie S5. A glide by gecko 1 viewed from the top camera.

Supplementary Material 9: Movie S6. A glide by gecko 2 viewed from the top camera.

Supplementary Material 10: Movie S7. A glide by gecko 2 viewed from the top camera.

Supplementary Material 11: Movie S8. A glide by gecko 3 viewed from the top camera.

Supplementary Material 12: Movie S9. A glide by gecko 5 viewed from the top camera.

Supplementary Material 13: Movie S10. A glide by gecko 5 viewed from the top camera.

Supplementary Material 14: Movie S11. A glide by gecko 6 viewed from the top camera.

Supplementary Material 15: Movie S12. A glide by gecko 9 viewed from the top camera.

Supplementary Material 16: Movie S13. A glide by gecko 10 viewed from the top camera.

Supplementary Material 17: Movie S14. A glide by gecko 10 viewed from the top camera.

Supplementary Material 18: Movie S15. A glide by gecko 1 viewed from the front camera.

Supplementary Material 19: Movie S16. A glide by gecko 6 viewed from the side camera.

Supplementary Material 20: Movie S17. A glide by gecko 10 viewed from the front camera.

Supplementary Material 21: Movie S18. A glide by gecko 10 viewed from the side camera.

## Data Availability

Data is provided within the manuscript or supplementary information files.
