# Supplementary material for: Forelimb motion and reciprocation mediate aerodynamic control in a gliding lizard
Source: BMC Ecol Evol. 2025 Nov 6;25:117. doi: 10.1186/s12862-025-02419-2 (PMC12590867; doi:10.1186/s12862-025-02419-2)
Supplement: Supplementary file 1 — Supplementary Material 1. [file 12862_2025_2419_MOESM1_ESM.docx]

**Supplemental Materials**

**Supplemental text:**

Study taxon and husbandry

All geckos underwent a 2–6-week initial quarantine period during which they were monitored to confirm good health. We began aerial performance trials immediately after the quarantine was terminated. Lizards were transported from husbandry rooms to experimental facilities by capturing a gecko by hand and placing it individually in a small plastic terrarium within which animals remained when not participating in experiments, except for placement of body and limb landmarks (see below), and for morphological measurements. Trials were separated by a minimum of 24 hours to provide rest.

To measure the gecko’s mass, we recorded the combined mass of the lizard and its terrarium. We then captured the gecko by hand, measured its snout-vent length, measured the mass of the terrarium absent the gecko (yielding by subtraction the gecko’s mass), and then applied landmarks to the gecko’s skin (Figure 1; Figure 2). While the gecko was restrained for mass measurement, we turned on the wind tunnel and allowed the airflow to reach the intended velocity. Immediately after each trial, we turned off the wind tunnel and returned the gecko to its terrarium.

Camera calibration

We used the detectCheckerboardPoints Matlab function (sigma of 3, whereby sigma is the bandwidth of the gaussian filter for corner detection; see Geiger et al., 2012 for details) for the stereo camera calibration. We filmed a 10 square-wide and 7 square-long checkerboard (square length of 16.33 mm) with all three cameras simultaneously, adjusting its orientation and position within the arena. The checkerboard occupied close to 25% of the field of view of each camera.

Extrinsic and intrinsic reconstruction parameters, as well as distortion coefficients of each camera were predicted using image stacks from these videos (Geiger et al., 2012). Extrinsic camera parameters included both rotation and translation of the global coordinate system to match the camera’s coordinate system. Intrinsic camera parameters included the focal length, optical center, and skew coefficient that related the two-dimensional images captured by each camera to its three-dimensional coordinate system. Cameras were focused several centimeters above the testing arena's floor at the nominal target height for filming aerial behavior.

Coordinate reconstruction

We used the ‘undistortPoints’ function in matlab to correct our tracked landmark coordinates for lens distortion in each camera used given the intrinsic and extrinsic camera parameters. We then used the triangulate function in Matlab to generate 3D locations of the undistorted points and their reprojection errors. The function reprojects each world point back onto both camera images and calculates the difference between the detected and reprojected points in the images.

To our understanding, there are limitations to calibrating three (but not two) cameras with differing frame sizes, so we used two-camera pairs (i.e., front and side, front and top, and side and top) to triangulate the 3D coordinates for analysis. To select the two-camera pair to use for analysis, we calculated the proportion of frames in which each landmark was visible from each camera in each trial. If any landmark was occluded in more than 40% of frames in only one camera, we selected the camera pair that excluded that camera. If multiple cameras had similar levels of landmark occlusion, we generated 3D coordinates with all camera pairs views and selected the pair with the lowest digital reprojection error.

Data selection

*Landmark selection* – Although we only used 14 landmarks for analyses, we also placed two unused points: on the head between the eyes and midway between the pelvis and tail tip. These were uninformative for our measurements, but they are present in the images and videos of the geckos.

*Trial selection* – Events for which the gecko was in contact with the arena walls or floor were excluded so as to eliminate possible contributions of contact forces to body displacement.

*Forelimb selection* – In selecting the forelimb to use, our priority was to select the forelimb that moved through the larger angle. However, we sought to capture the entire sweep of shoulder retraction, so if only one forelimb had a local minimum prior to the sweep, we selected that forelimb over the other. If neither forelimb started at a local minimum, we selected the limb with the larger starting angle.

Wind tunnel

The wind tunnel consisted of a rigid plastic cylinder (diameter of ~ 71 cm), at the base of which was positioned a rotary fan that generated upward airflow. Two layers of mesh and a 10 cm layer of metal honeycomb (diagonal of ~5 mm) were positioned above the fan to reduce turbulence. A square-shaped contracting funnel was fastened atop the cylinder to increase wind speed and to make flow more uniform in cross-section.

The flight arena was positioned above this contracting funnel (width of 30.5 cm, length of 30.5 cm), and was bounded on four sides by transparent 5 mm thick acrylic walls. Two of these walls (height: 50.8 cm) were tall so to increase vertically the spatial volume within which geckos could be filmed, whereas the other two walls (height of 25.4 cm) were shorter to allow access to the inside of the arena. The base of the arena consisted of a cloth mesh (elliptical holes with major axis length of 4 mm and minor axis length of 2.5 mm) below a layer of wire mesh (2.54 cm squares). Mosquito netting was fastened to the top edge of the walls and was extended upward to surround a camera positioned above the floor of the arena. The net was sealed above and laterally to prevent escape of the geckos, but a small gap (length of 15 cm) was left unsealed above the two shorter walls to enable arena access.

Spatial variation of the wind tunnel was assessed by partitioning the bottom of the flight arena into 144 squares, each one being 2.54 cm in length, and by then measuring wind speed above each square (at a nominal wind speed in the working section of ~9.0 m/s, which was about 13% higher than 8.0 m/s, the mean speed setting at which the geckos typically glided). Speed at each square (excepting two squares in the corners) was within 25% of the overall mean among all squares (8.41 m/s) and was thus similar to the spatial variation found in tropical forests (McCay, 2003). Furthermore, the mean windspeed used for biomechanical analyses was approximately 7% faster than the average speed among all 144 squares, as it was measured at the center of the arena where speeds tended to be fastest. Speed variation across the working section of the tunnel was also typical of naturally occurring spatial variation along vertical gradients within forest canopies (see Brown et al., 2022a).

For biomechanical analyses, we determined the mean wind speed at the center of the wind tunnel (as measured using a hand-held anemometer; VelociCalc model #8346, TSI Inc., Shoreview, MN, USA) at the nominal target height for filming. Mean wind speed was estimated over time by averaging three instantaneous wind speeds. Instantaneous wind speeds were measured by holding the probe in the airstream for thirty seconds and recording the averaged wind speed over 10, 20, and 30 second periods. We tailored the wind speed for each gecko by matching the geckos equilibrium velocity as estimated during preliminary trials to the mean wind speed.

Kinematics measurements details

*Roll -* To calculate body roll, we constructed a line between the shoulder landmarks (i.e., the shoulder axis), and generated the midpoint of the shoulder axis (i.e., the midshoulder). We then created a vector (parallel to the longitudinal body axis) between the midshoulder and midbody points and computationally rotated all positional data such that the body axis was aligned with the global x-axis. We considered roll to be the angle between the vector normal to the rotated chest plane and the global z-axis, such that a roll to the gecko’s right was represented by a negative angle, and a roll to the left was positive.

*Pitch –* To calculate body pitch, we computationally rotated the gecko’s original smoothed positional data such that the shoulder axis aligned with the y-axis. Pitch was then calculated as the angle between the vector normal to the chest plane and the z-axis; positive pitch angle represents nose-up pitch, and negative pitch is a nose-down orientation.

*Bend –* We approximated body curvature by calculating the bend angle of the body at the midbody point (termed body bend). We identified a midpoint between the hip points (midhip) and projected it onto the chest plane (projected midhip). We added the angle bound by these three points (with its vertex at midbody) to 180° and considered this body bend angle. With this configuration, body bend angles less than 180° represent a flexed vertebral column (i.e., with hips below the chest plane) and angles more than 180° represent vertebral extension (i.e., with hips above the chest plane).

*Shoulder adduction* – To estimate the left shoulder adduction angle, we calculated the distance between the left elbow point and the chest plane, and the length of the line between the left shoulder and the left elbow (i.e., corresponding to the humerus). We then used trigonometry to estimate the angle between the humerus and the chest plane. Negative and positive angles represent ventral and dorsal positions, respectively. We repeated this procedure for the right shoulder.

*Shoulder retraction –* We computed the shoulder retraction angle by projecting the left elbow point onto the chest plane, thus creating a vector between the left shoulder and the projected left elbow, and then calculated the angle between this vector and the shoulder axis such that a positive shoulder retraction angle represents an anterior position, and a negative angle represents a posterior position. We repeated this procedure for the right shoulder.

*Hip adduction and retraction* – We used the same method as with shoulder adduction and shoulder retraction, using the left hip, the right hip, and the midbody to define an abdominal plane instead of the shoulders and chest plane.

*Elbow flexion –* To estimate elbow flexion angles, we calculated the 3D angle between the humerus and the vector defined by the elbow and the wrist for the right and left forelimbs.

*Knee flexion* – Calculation of knee flexion is the same as elbow flexion but replacing the humerus, elbow, and wrist with the femur, knee, and angle, respectively.

*Tail* – To evaluate tail orientation, we treated the tail as a rigid rod. To compute the angle of the tail on the frontal plane, we projected the tail tip onto the abdominal plane and created a vector between midhip and the projected tail tip. We considered tail angle in the frontal plane to be the angle between this vector and the abdominal axis (i.e., the vector between midbody and midhip). To calculate the angle of the tail in the sagittal plane, we rotated positional data such that the abdominal axis was parallel to the x-axis, and the abdominal plane was parallel to the (x,y) plane. We then projected the tail tip onto the (x,z) plane, and calculated the angle between the x-axis and the line between midhip and the projected tail tip.

*Velocities and accelerations –* For velocity estimates, we used the x-, y-, and z-components of the midbody velocity (calculated from the first derivative of the spline function used to smooth positional data for each trial) because it was the landmark nearest to the center of body mass (i.e., approximately midway between the forelimbs and hindlimbs; see Autumn et al., 2006). Vertical velocity was simply the z-component of body velocity. To calculate horizontal speed, we used the square root of the sum of the squares of the x- and y-components, according to the Pythagorean theorem. We then used the horizontal speed to calculate forward velocity, defined here as the horizontal speed in the direction of the gecko’s body axis such that a positive forward velocity was in the gecko’s cranial direction. We first projected the body axis of the gecko onto the (x,y) plane and rotated it such that the projected body axis was parallel to the x-axis. We also applied this rotation to the horizontal speed vector and considered the forward velocity to be the x-component of the projected horizontal speed. We estimated glide angle as the angle between the vertical velocity vector and the horizontal speed vector, and considered forward velocity, vertical velocity, and glide angle as primary measures of glide performance. Similarly, we used the x-, y-, and z-components of midbody acceleration (as calculated from the second derivative of the spline function smoothing positional data) for all acceleration estimates, calculated analogously to the velocity estimates, and obtained values for vertical, horizontal, and forward acceleration.
