# Supplementary material for: Forelimb motion and reciprocation mediate aerodynamic control in a gliding lizard
Source: BMC Ecol Evol. 2025 Nov 6;25:117. doi: 10.1186/s12862-025-02419-2 (PMC12590867; doi:10.1186/s12862-025-02419-2)
Supplement: Supplementary file 2 — Supplementary Material 2. [file 12862_2025_2419_MOESM2_ESM.docx]

**Supplemental Figures:**

**
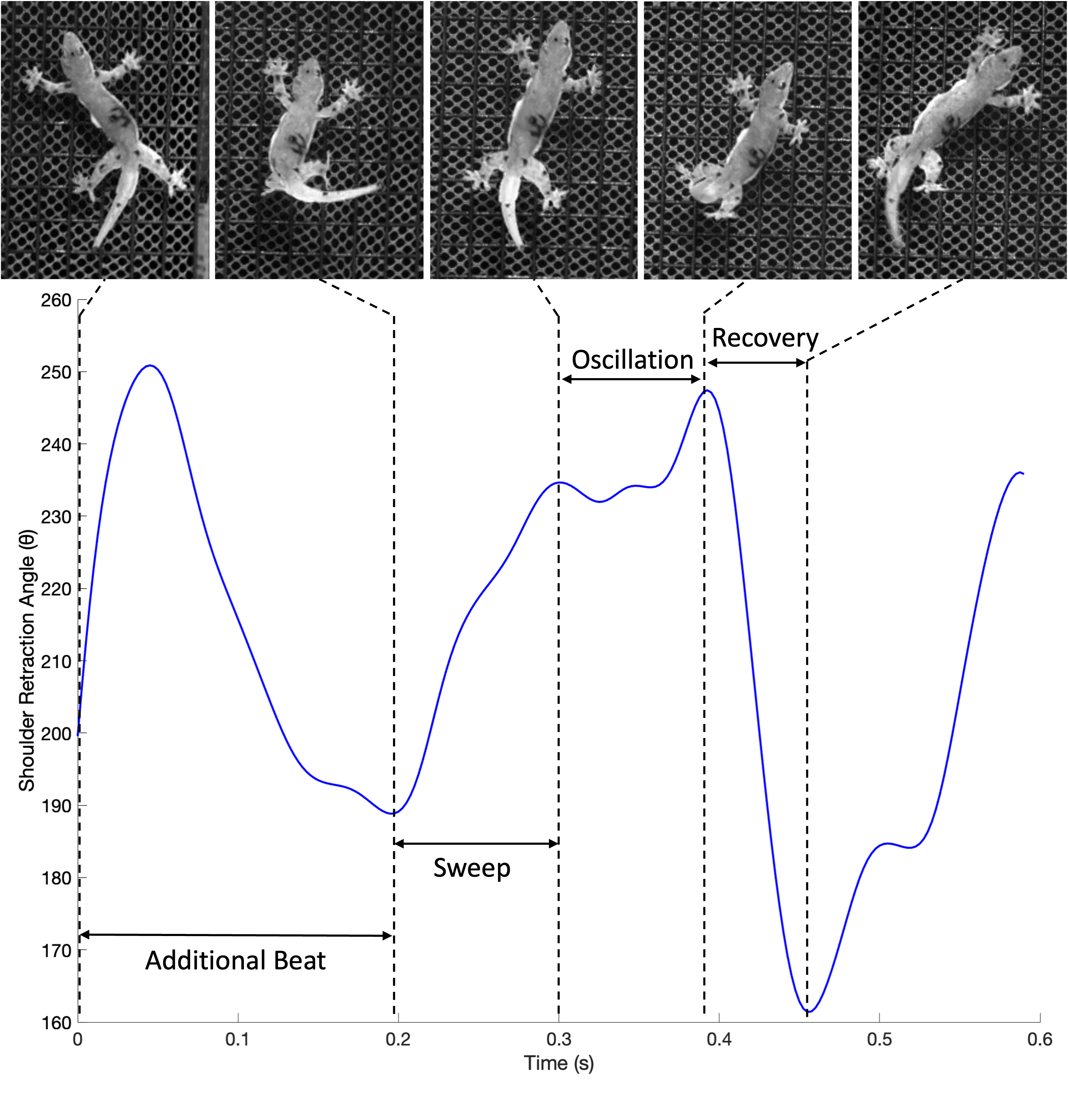
Figure S1.** **Representative trace of** **shoulder retraction behavior variation.** An additional beat, a sweep, oscillations in the swept configuration posture, and a recovery stroke are present. The data derive from trial 09 from gecko #3.

**
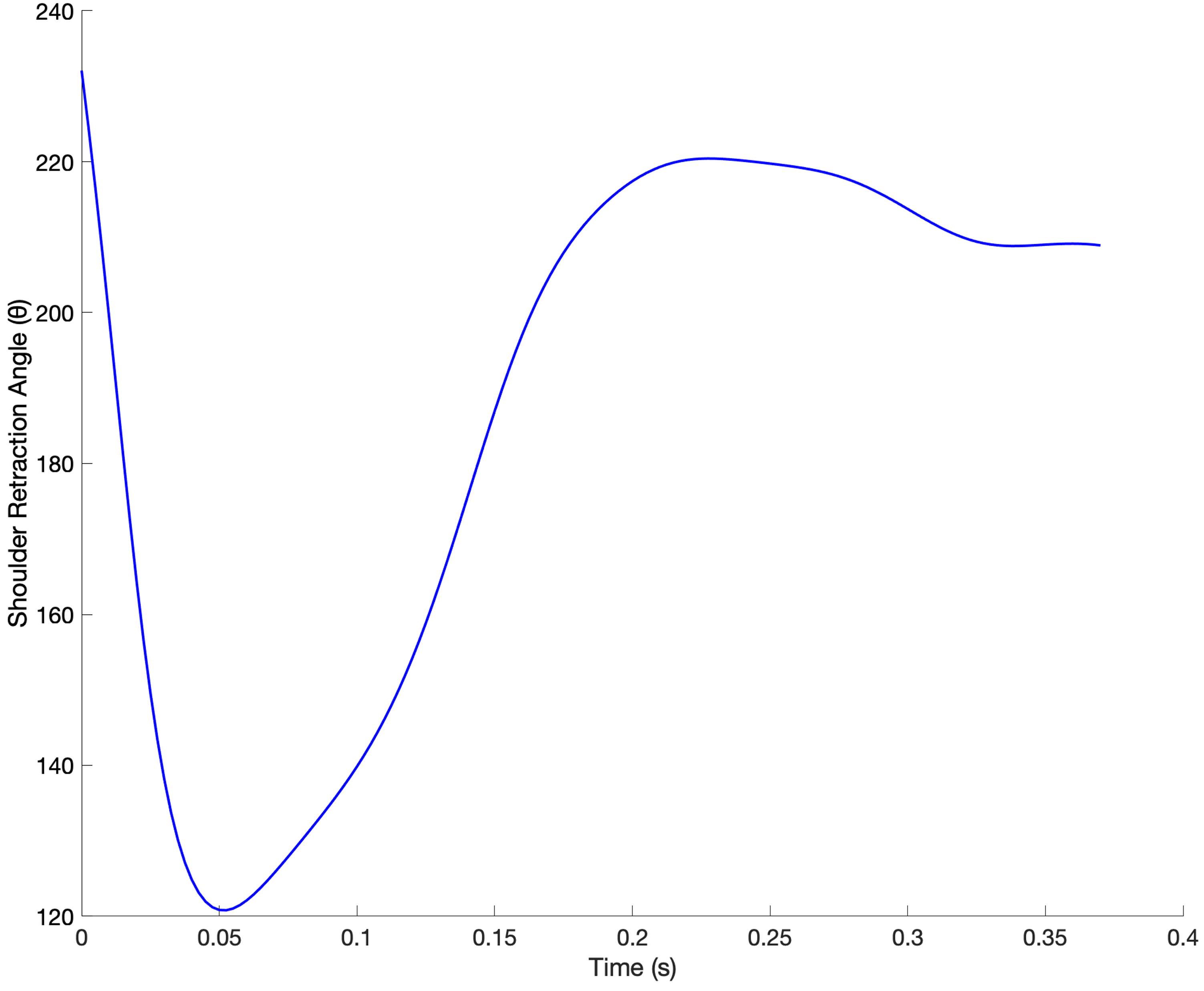
Figure S2. Trace of** **shoulder retraction** wherein the local maximum occurred prior to the onset of data collection. The data represents gecko 5’s trial 08.

**Figure S3. Representative cross-correlation** between shoulder retraction angle and forward velocity. The x-axis shows the lag of variable 2 (forward velocity) relative to variable 1 (shoulder retraction angle). The y-axis shows correlation coefficient from the cross-correlation test. The maximum correlation coefficient for this example occurred at a lag of 12 frames (i.e., 0.03 s). A Pearson Correlation test on the lagged data showed a significant positive correlation (p > 0.001, R^2^ = 0.86).

**
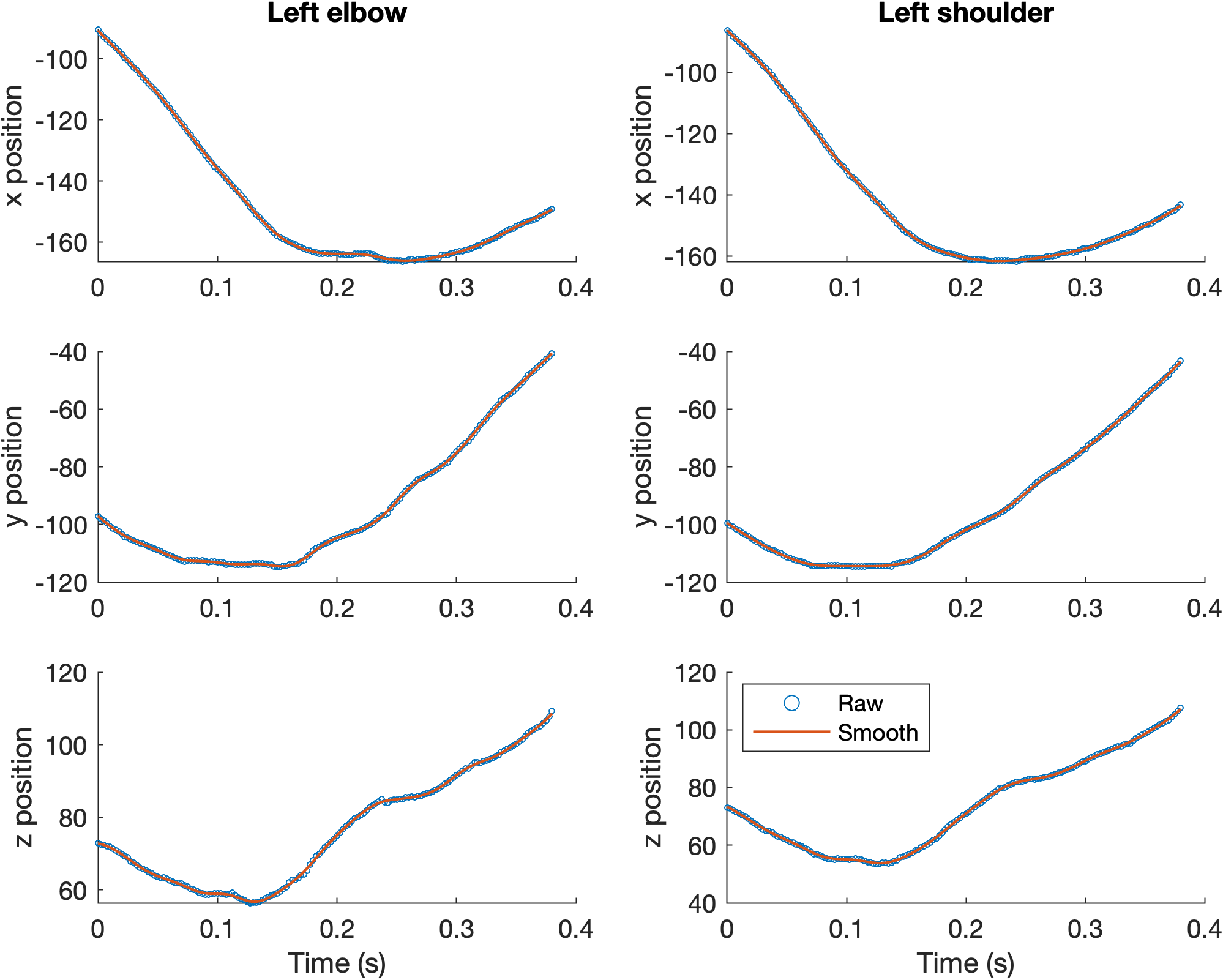
Figure S4.** **Representative smoothing splines** for each axial direction in the global coordinate system for two landmarks. The smoothed data closely follows the raw data while eliminating noise. The data represents gecko 1’s trial 03.
