# Supplementary material for: Forelimb motion and reciprocation mediate aerodynamic control in a gliding lizard
Source: BMC Ecol Evol. 2025 Nov 6;25:117. doi: 10.1186/s12862-025-02419-2 (PMC12590867; doi:10.1186/s12862-025-02419-2)
Supplement: Supplementary file 3 — Supplementary Material 3. [file 12862_2025_2419_MOESM3_ESM.docx]

**Supplemental Tables:**

|  | Sky-Swept Comparison  (n = 20) | | Sky-Swept Comparison  (n = 9) | | |
| --- | --- | --- | --- | --- | --- |
| Variable | Sky Lilliefors p | Swept Lilliefors p | Sky Lilliefors p | Swept Lilliefors p | Recover Lilliefors p |
| Roll (°) | 0.229 | 0.155 | 0.324 | 0.027 | 0.241 |
| Pitch (°) | 0.500 | 0.500 | 0.478 | 0.241 | 0.471 |
| Bend (°) | 0.500 | 0.500 | 0.5 | 0.5 | 0.491 |
| Shoulder retraction (°) | 0.500 | 0.500 | 0.153 | 0.223 | 0.5 |
| Shoulder adduction (°) | 0.164 | 0.360 | 0.064 | 0.037 | 0.353 |
| Elbow flexion (°) | 0.500 | 0.093 | 0.5 | 0.085 | 0.5 |
| Hip retraction (°) | 0.278 | 0.500 | 0.341 | 0.5 | 0.5 |
| Hip adduction (°) | 0.390 | 0.500 | 0.381 | 0.424 | 0.5 |
| Knee flexion (°) | 0.500 | 0.472 | 0.5 | 0.456 | 0.281 |
| Wrist shoulder adduction (°) | 0.036 | 0.378 | 0.5 | 0.392 | 0.5 |
| Tail sagittal (°) | 0.073 | 0.500 | 0.24 | 0.5 | 0.5 |
| Tail frontal (°) | 0.100 | 0.211 | 0.039 | 0.251 | 0.5 |
| Vertical velocity (m/s) | 0.500 | 0.500 | 0.5 | 0.5 | 0.5 |
| Horizontal speed (m/s) | 0.221 | 0.163 | 0.5 | 0.474 | 0.165 |
| Forward velocity (m/s) | 0.500 | 0.500 | 0.5 | 0.375 | 0.5 |
| Glide angle (°) | 0.087 | 0.074 | 0.5 | 0.387 | 0.1 |
| Vertical acceleration (m/s^2^) | 0.500 | 0.366 | 0.5 | 0.095 | 0.453 |
| Horizontal acceleration (m/s^2^) | 0.123 | 0.500 | 0.049 | 0.5 | 0.202 |
| Forward acceleration (m/s^2^) | 0.500 | 0.500 | 0.11 | 0.5 | 0.019 |

**Table S1.**

P-values from the Lilliefors tests of normality within each posture for each of the variables. P-values indicating a statistically significant correlation are in boldface.

Table S2.

| ID | Trial | Value | Shoulder Retraction-Shoulder Adduction | Shoulder Retraction-Bend | Shoulder Retraction-Pitch | Shoulder Retraction-Forward Velocity | Shoulder Retraction-Vertical Velocity | Shoulder Retraction  -Forward Acceleration | Shoulder Retraction  -Vertical Acceleration | Bend  - Pitch |
| --- | --- | --- | --- | --- | --- | --- | --- | --- | --- | --- |
| 1 | 03A | Lag | 0.113 | 0.008 | 0.070 | 0.000 | 0.135 | 0.103 | 0.030 | 0.0575 |
|  |  | p | **< 0.001** | **< 0.001** | **< 0.001** | **< 0.001** | **< 0.001** | **< 0.001** | **< 0.001** | **< 0.001** |
|  |  | R^2 | -0.84 | -0.86 | -0.83 | 0.40 | -0.93 | 0.81 | 0.85 | 0.82 |
| 1 | 03B | Lag | 0.000 | 0.010 | 0.060 | 0.000 | 0.000 | 0.000 | 0.098 | 0.005 |
|  |  | p | **< 0.001** | **< 0.001** | 0.257 | **< 0.001** | **< 0.001** | **0.049** | 0.285 | **< 0.001** |
|  |  | R^2 | 0.30 | -0.78 | 0.10 | 0.29 | 0.40 | 0.16 | 0.10 | 0.64 |
| 1 | 11A | Lag | 0.005 | 0.000 | 0.003 | 0.000 | 0.000 | 0.000 | 0.000 | 0.0575 |
|  |  | p | **< 0.001** | **< 0.001** | **< 0.001** | **< 0.001** | **0.002** | **< 0.001** | **< 0.001** | **< 0.001** |
|  |  | R^2 | 0.59 | -0.62 | -0.89 | 0.74 | 0.31 | 0.93 | 0.80 | 0.89 |
| 2 | 05B | Lag | 0.100 | 0.000 | 0.025 | 0.000 | 0.000 | 0.000 | 0.035 | 0.015 |
|  |  | p | **< 0.001** | **< 0.001** | **< 0.001** | **< 0.001** | **< 0.001** | **< 0.001** | **< 0.001** | **< 0.001** |
|  |  | R^2 | -0.73 | -0.89 | 0.93 | 0.88 | 0.96 | -0.98 | 0.95 | -0.88 |
| 2 | 07B | Lag | 0.000 | 0.033 | 0.048 | 0.030 | 0.000 | 0.035 | 0.023 | 0 |
|  |  | p | **< 0.001** | **< 0.001** | **< 0.001** | **< 0.001** | **< 0.001** | **< 0.001** | **< 0.001** | **< 0.001** |
|  |  | R^2 | -0.45 | -0.92 | -0.86 | 0.86 | 0.65 | 0.97 | 0.91 | 0.9 |
| 3 | 09X | Lag | 0.000 | 0.075 | 0.140 | 0.070 | 0.005 | 0.128 | 0.030 | 0.0775 |
|  |  | p | **< 0.001** | **< 0.001** | **< 0.001** | **< 0.001** | **< 0.001** | **< 0.001** | **0.006** | **< 0.001** |
|  |  | R^2 | 0.57 | -0.56 | -0.84 | 0.31 | 0.26 | 0.86 | 0.18 | 0.72 |
| 3 | 11A | Lag | 0.000 | 0.000 | 0.075 | 0.000 | 0.000 | 0.000 | 0.000 | 0.1475 |
|  |  | p | **< 0.001** | **< 0.001** | **< 0.001** | **< 0.001** | **< 0.001** | **< 0.001** | **< 0.001** | **< 0.001** |
|  |  | R^2 | -0.88 | -0.92 | 0.55 | 0.83 | 0.89 | 0.80 | 0.68 | -0.85 |
| 3 | 11B | Lag | 0.000 | 0.035 | 0.000 | 0.063 | 0.060 | 0.058 | 0.020 | 0.03 |
|  |  | p | **< 0.001** | **< 0.001** | **< 0.001** | **< 0.001** | **< 0.001** | **< 0.001** | **< 0.001** | **< 0.001** |
|  |  | R^2 | 0.70 | -0.94 | 0.60 | -0.81 | -0.87 | 0.88 | 0.74 | 0.93 |
| 4 | 11X | Lag | 0.000 | 0.048 | 0.040 | 0.035 | 0.028 | 0.053 | 0.035 | 0 |
|  |  | p | **< 0.001** | **< 0.001** | **< 0.001** | **< 0.001** | **< 0.001** | **< 0.001** | **< 0.001** | **< 0.001** |
|  |  | R^2 | -0.88 | 0.80 | -0.84 | -0.74 | -0.83 | -0.72 | -0.90 | -0.9 |
| 5 | 03X | Lag | 0.005 | 0.008 | 0.015 | 0.000 | 0.043 | 0.053 | 0.008 | 0 |
|  |  | p | **< 0.001** | **< 0.001** | **0.038** | 0.437 | **< 0.001** | **< 0.001** | **0.010** | **< 0.001** |
|  |  | R^2 | -0.65 | -0.43 | -0.19 | -0.07 | -0.40 | 0.63 | 0.23 | -0.76 |
| 5 | 06X | Lag | 0.000 | 0.118 | 0.000 | 0.000 | 0.090 | 0.075 | 0.000 | 0.005 |
|  |  | p | **< 0.001** | **< 0.001** | **< 0.001** | **< 0.001** | **< 0.001** | **< 0.001** | **< 0.001** | **< 0.001** |
|  |  | R^2 | -0.42 | -0.94 | -0.62 | 0.71 | 0.92 | -0.78 | -0.70 | -0.61 |
| 5 | 08X | Lag | 0.000 | 0.008 | 0.038 | 0.013 | 0.010 | 0.035 | 0.028 | 0.055 |
|  |  | p | **< 0.001** | **< 0.001** | **< 0.001** | **< 0.001** | **< 0.001** | **< 0.001** | **< 0.001** | **< 0.001** |
|  |  | R^2 | 0.49 | -0.93 | -0.90 | 0.85 | 0.80 | 0.94 | 0.97 | 0.86 |
| 5 | 10B | Lag | 0.010 | 0.003 | 0.000 | 0.000 | 0.043 | 0.078 | 0.168 | 0.0175 |
|  |  | p | 0.054 | 0.894 | **< 0.001** | **0.008** | **< 0.001** | **< 0.001** | **< 0.001** | **< 0.001** |
|  |  | R^2 | 0.15 | 0.01 | 0.57 | -0.20 | -0.44 | -0.59 | -0.77 | -0.78 |
| 6 | 04A | Lag | 0.000 | 0.040 | 0.000 | 0.013 | 0.100 | 0.000 | 0.023 | 0.09 |
|  |  | p | **< 0.001** | **< 0.001** | **< 0.001** | **< 0.001** | **< 0.001** | **< 0.001** | **< 0.001** | **< 0.001** |
|  |  | R^2 | 0.76 | -0.74 | 0.90 | 0.57 | -0.87 | -0.33 | 0.90 | 0.74 |
| 6 | 04B | Lag | 0.000 | 0.048 | 0.000 | 0.000 | 0.000 | 0.100 | 0.053 | 0.0775 |
|  |  | p | **< 0.001** | **< 0.001** | 0.767 | **< 0.001** | **< 0.001** | **< 0.001** | **< 0.001** | **< 0.001** |
|  |  | R^2 | 0.69 | -0.91 | -0.02 | 0.77 | 0.83 | 0.93 | 0.94 | 0.83 |
| 9 | 07A | Lag | 0.033 | 0.038 | 0.000 | 0.000 | 0.000 | 0.000 | 0.025 | 0.0075 |
|  |  | p | **< 0.001** | **< 0.001** | **< 0.001** | **< 0.001** | **< 0.001** | **< 0.001** | **< 0.001** | **< 0.001** |
|  |  | R^2 | 0.82 | -0.62 | 0.76 | 0.70 | 0.95 | -0.97 | 0.91 | -0.6 |
| 9 | 07B | Lag | 0.035 | 0.000 | 0.020 | 0.000 | 0.000 | 0.000 | 0.000 | 0.0075 |
|  |  | p | **0.015** | **< 0.001** | **< 0.001** | **< 0.001** | **< 0.001** | **< 0.001** | **< 0.001** | 0.597 |
|  |  | R^2 | 0.37 | -0.71 | 0.64 | 0.75 | 0.79 | -0.91 | -0.68 | -0.07 |
| 10 | 06X | Lag | 0.040 | 0.000 | 0.000 | 0.000 | 0.000 | 0.065 | 0.000 | 0 |
|  |  | p | **< 0.001** | **< 0.001** | **< 0.001** | **< 0.001** | **< 0.001** | **< 0.001** | **< 0.001** | **< 0.001** |
|  |  | R^2 | 0.81 | -0.96 | 0.79 | 0.88 | 0.85 | 0.66 | 0.96 | -0.85 |
| 10 | 09A | Lag | 0.000 | 0.000 | 0.000 | 0.000 | 0.093 | 0.000 | 0.038 | 0 |
|  |  | p | **< 0.001** | **< 0.001** | **< 0.001** | **< 0.001** | **< 0.001** | **< 0.001** | **< 0.001** | **< 0.001** |
|  |  | R^2 | 0.39 | -0.84 | 0.92 | -0.58 | -0.92 | -0.80 | 0.90 | -0.92 |
| 10 | 09B | Lag | 0.015 | 0.028 | 0.000 | 0.020 | 0.000 | 0.023 | 0.020 | 0.0525 |
|  |  | p | **< 0.001** | **< 0.001** | **< 0.001** | **< 0.001** | **< 0.001** | **< 0.001** | **< 0.001** | **< 0.001** |
|  |  | R^2 | 0.92 | -0.90 | 0.73 | 0.86 | 0.67 | 0.82 | 0.89 | 0.69 |

Results from the cross-correlations for each of the indicated variable pairs. The sign of the R^2^ value indicates negative and positive correlations. P-values indicating a statistically significant correlation are in boldface. All lag values are in seconds.

Table S3.

| ID | Trial | Value | Pitch-Forward Velocity | Pitch-Vertical velocity | Pitch-Forward Acceleration | Pitch-  Vertical Acceleration | Bend-Forward Velocity | Bend-Vertical Velocity | Bend-Forward Acceleration | Bend-  Vertical Acceleration |
| --- | --- | --- | --- | --- | --- | --- | --- | --- | --- | --- |
| 1 | 03A | Lag | 0.085 | 0.068 | 0.050 | 0.000 | 0.000 | 0.000 | 0.000 | 0.033 |
|  |  | p | **< 0.001** | **< 0.001** | **< 0.001** | **< 0.001** | **< 0.001** | **< 0.001** | **< 0.001** | **< 0.001** |
|  |  | R^2 | 0.76 | 0.67 | -0.85 | -0.54 | -0.74 | -0.61 | 0.53 | -0.68 |
| 1 | 03B | Lag | 0.000 | 0.000 | 0.028 | 0.003 | 0.043 | 0.048 | 0.008 | 0.053 |
|  |  | p | **< 0.001** | **< 0.001** | **0.008** | **< 0.001** | **< 0.001** | **< 0.001** | 0.314 | **< 0.001** |
|  |  | R^2 | 0.68 | 0.69 | -0.22 | 0.63 | 0.65 | 0.73 | 0.08 | 0.48 |
| 1 | 11A | Lag | 0.085 | 0.045 | 0.000 | 0.000 | 0.000 | 0.000 | 0.018 | 0.000 |
|  |  | p | **< 0.001** | **< 0.001** | **< 0.001** | **< 0.001** | **< 0.001** | **< 0.001** | **< 0.001** | **< 0.001** |
|  |  | R^2 | 0.91 | 0.88 | -0.71 | -0.49 | -0.96 | -0.91 | -0.92 | -0.95 |
| 2 | 05B | Lag | 0.010 | 0.000 | 0.000 | 0.015 | 0.000 | 0.000 | 0.000 | 0.020 |
|  |  | p | **< 0.001** | **< 0.001** | **< 0.001** | **< 0.001** | **< 0.001** | **< 0.001** | **< 0.001** | **< 0.001** |
|  |  | R^2 | 0.78 | 0.55 | -0.81 | 0.91 | -0.89 | -0.83 | 0.96 | -0.96 |
| 2 | 07B | Lag | 0.000 | 0.000 | 0.000 | 0.000 | 0.000 | 0.000 | 0.000 | 0.000 |
|  |  | p | **< 0.001** | **< 0.001** | **< 0.001** | **< 0.001** | **< 0.001** | **< 0.001** | **< 0.001** | **< 0.001** |
|  |  | R^2 | -0.86 | -0.66 | -0.89 | -0.83 | -0.93 | -0.80 | -0.98 | -0.98 |
| 3 | 09X | Lag | 0.098 | 0.118 | 0.000 | 0.043 | 0.035 | 0.028 | 0.060 | 0.080 |
|  |  | p | **< 0.001** | **< 0.001** | **< 0.001** | **< 0.001** | **< 0.001** | **< 0.001** | **< 0.001** | **< 0.001** |
|  |  | R^2 | 0.83 | -0.75 | -0.96 | 0.71 | 0.23 | -0.28 | -0.74 | 0.72 |
| 3 | 11A | Lag | 0.050 | 0.063 | 0.035 | 0.128 | 0.000 | 0.000 | 0.000 | 0.000 |
|  |  | p | **< 0.001** | **< 0.001** | **< 0.001** | **< 0.001** | **< 0.001** | **< 0.001** | **< 0.001** | **< 0.001** |
|  |  | R^2 | -0.85 | -0.91 | -0.83 | -0.81 | -0.83 | -0.89 | -0.77 | -0.85 |
| 3 | 11B | Lag | 0.000 | 0.000 | 0.000 | 0.025 | 0.015 | 0.015 | 0.010 | 0.000 |
|  |  | p | **< 0.001** | **< 0.001** | **< 0.001** | **0.011** | **< 0.001** | **< 0.001** | **< 0.001** | **< 0.001** |
|  |  | R^2 | 0.68 | 0.71 | -0.68 | 0.33 | 0.85 | 0.90 | -0.89 | -0.77 |
| 4 | 11X | Lag | 0.000 | 0.000 | 0.000 | 0.000 | 0.000 | 0.000 | 0.000 | 0.000 |
|  |  | p | **< 0.001** | **< 0.001** | **< 0.001** | **< 0.001** | **< 0.001** | **< 0.001** | **< 0.001** | **< 0.001** |
|  |  | R^2 | 0.97 | 0.90 | 0.93 | 0.96 | -0.83 | -0.64 | -0.92 | -0.75 |
| 5 | 03X | Lag | 0.000 | 0.000 | 0.125 | 0.000 | 0.000 | 0.000 | 0.068 | 0.005 |
|  |  | p | **< 0.001** | **< 0.001** | **< 0.001** | **< 0.001** | **< 0.001** | **< 0.001** | **< 0.001** | **< 0.001** |
|  |  | R^2 | 0.95 | 0.88 | 0.97 | 0.86 | -0.81 | -0.56 | -0.80 | -0.96 |
| 5 | 06X | Lag | 0.000 | 0.068 | 0.000 | 0.000 | 0.010 | 0.053 | 0.043 | 0.008 |
|  |  | p | **< 0.001** | **< 0.001** | **< 0.001** | **< 0.001** | **0.019** | **< 0.001** | **< 0.001** | **< 0.001** |
|  |  | R^2 | -0.83 | -0.96 | -0.42 | 0.95 | 0.25 | 0.93 | -0.86 | -0.56 |
| 5 | 08X | Lag | 0.000 | 0.000 | 0.000 | 0.000 | 0.000 | 0.000 | 0.050 | 0.038 |
|  |  | p | **< 0.001** | **< 0.001** | **< 0.001** | **< 0.001** | **< 0.001** | **< 0.001** | **< 0.001** | **< 0.001** |
|  |  | R^2 | -0.62 | -0.57 | -0.94 | -0.86 | -0.93 | -0.90 | -0.91 | -0.94 |
| 5 | 10B | Lag | 0.058 | 0.000 | 0.075 | 0.003 | 0.000 | 0.000 | 0.000 | 0.000 |
|  |  | p | **< 0.001** | **< 0.001** | **< 0.001** | **< 0.001** | **< 0.001** | **< 0.001** | **< 0.001** | **< 0.001** |
|  |  | R^2 | 0.80 | 0.54 | -0.94 | 0.83 | -0.88 | -0.69 | 0.67 | -0.96 |
| 6 | 04A | Lag | 0.033 | 0.103 | 0.005 | 0.033 | 0.050 | 0.058 | 0.035 | 0.000 |
|  |  | p | **< 0.001** | **< 0.001** | **< 0.001** | **< 0.001** | **0.007** | **< 0.001** | **< 0.001** | **< 0.001** |
|  |  | R^2 | 0.54 | -0.97 | -0.44 | 0.87 | -0.22 | 0.66 | -0.53 | -0.50 |
| 6 | 04B | Lag | 0.000 | 0.000 | 0.073 | 0.048 | 0.000 | 0.000 | 0.048 | 0.000 |
|  |  | p | **< 0.001** | **0.006** | **< 0.001** | **< 0.001** | **< 0.001** | **< 0.001** | **< 0.001** | **< 0.001** |
|  |  | R^2 | -0.38 | -0.22 | 0.94 | 0.76 | -0.30 | -0.27 | -0.80 | -0.81 |
| 9 | 07A | Lag | 0.075 | 0.000 | 0.000 | 0.015 | 0.000 | 0.000 | 0.000 | 0.013 |
|  |  | p | **< 0.001** | **< 0.001** | **< 0.001** | **< 0.001** | 0.124 | **0.016** | **< 0.001** | **< 0.001** |
|  |  | R^2 | 0.95 | 0.67 | -0.84 | 0.93 | -0.13 | -0.21 | 0.39 | -0.65 |
| 9 | 07B | Lag | 0.020 | 0.020 | 0.023 | 0.015 | 0.000 | 0.000 | 0.000 | 0.000 |
|  |  | p | **< 0.001** | **< 0.001** | **< 0.001** | **< 0.001** | **< 0.001** | **< 0.001** | **< 0.001** | **< 0.001** |
|  |  | R^2 | -0.95 | -0.95 | 0.96 | 0.89 | -0.96 | -0.96 | 0.93 | 0.87 |
| 10 | 06X | Lag | 0.000 | 0.000 | 0.000 | 0.000 | 0.000 | 0.000 | 0.000 | 0.000 |
|  |  | p | **< 0.001** | **< 0.001** | **< 0.001** | **< 0.001** | **< 0.001** | **< 0.001** | **< 0.001** | **< 0.001** |
|  |  | R^2 | 0.97 | 0.98 | -0.68 | 0.90 | -0.95 | -0.93 | 0.55 | -0.99 |
| 10 | 09A | Lag | 0.000 | 0.108 | 0.000 | 0.050 | 0.000 | 0.118 | 0.000 | 0.055 |
|  |  | p | **< 0.001** | **< 0.001** | **< 0.001** | **< 0.001** | **< 0.001** | **< 0.001** | **< 0.001** | **< 0.001** |
|  |  | R^2 | -0.53 | -0.96 | -0.77 | 0.81 | 0.45 | 0.94 | 0.71 | -0.73 |
| 10 | 09B | Lag | 0.030 | 0.000 | 0.058 | 0.040 | 0.000 | 0.065 | 0.000 | 0.000 |
|  |  | p | **< 0.001** | **< 0.001** | **< 0.001** | **< 0.001** | **< 0.001** | **< 0.001** | **< 0.001** | **< 0.001** |
|  |  | R^2 | 0.87 | 0.93 | 0.89 | 0.84 | -0.88 | 0.93 | -0.91 | -0.92 |

Results from the cross-correlations for each of the indicated variable pairs. R^2^ value indicates negative and positive correlations. P-values indicating a statistically significant correlation are in boldface. All lag values are in seconds.

**Table S4.**

| ID | Trial | Value | Shouler retraction-tail sagittal | Pitch - tail sagittal | Bend - tail sagittal |
| --- | --- | --- | --- | --- | --- |
| 01 | 03A | Lag | 0.06 | 0 | 0.06 |
|  |  | p | **p < 0.001** | **p < 0.001** | **p < 0.001** |
|  |  | R2 | -0.75 | 0.77 | 0.73 |
| 01 | 03B | Lag | 0 | 0 | 0.07 |
|  |  | p | **p < 0.001** | **0.005** | **p < 0.001** |
|  |  | R2 | -0.8 | -0.22 | -0.8 |
| 01 | 11A | Lag | 0 | 0 | 0.0375 |
|  |  | p | **p < 0.001** | **p < 0.001** | **p < 0.001** |
|  |  | R2 | -0.93 | 0.9 | 0.89 |
| 02 | 05B | Lag | 0.01 | 0 | 0 |
|  |  | p | **p < 0.001** | **p < 0.001** | **p < 0.001** |
|  |  | R2 | -0.57 | -0.59 | 0.64 |
| 02 | 07B | Lag | 0.005 | 0.1175 | 0 |
|  |  | p | **p < 0.001** | **p < 0.001** | **p < 0.001** |
|  |  | R2 | -0.9 | -0.73 | 0.65 |
| 03 | 09X | Lag | 0.0175 | 0.055 | 0 |
|  |  | p | **p < 0.001** | 0.07 | **p < 0.001** |
|  |  | R2 | 0.24 | -0.12 | 0.25 |
| 03 | 11A | Lag | 0 | 0 | 0 |
|  |  | p | **p < 0.001** | 0.056 | **p < 0.001** |
|  |  | R2 | -0.7 | -0.17 | 0.84 |
| 03 | 11B | Lag | 0.0075 | 0.04 | 0 |
|  |  | p | **p < 0.001** | **p < 0.001** | **p < 0.001** |
|  |  | R2 | -0.85 | -0.84 | 0.65 |
| 04 | 11X | Lag | 0.0075 | 0.0075 | 0 |
|  |  | p | **0.001** | **0.007** | **0.01** |
|  |  | R2 | 0.38 | 0.32 | -0.3 |
| 05 | 03X | Lag | 0 | 0 | 0 |
|  |  | p | 0.071 | **0.003** | 0.064 |
|  |  | R2 | -0.16 | 0.26 | -0.17 |
| 05 | 06X | Lag | 0.0025 | 0.015 | 0 |
|  |  | p | **p < 0.001** | **p < 0.001** | **p < 0.001** |
|  |  | R2 | 0.47 | -0.51 | 0.72 |
| 05 | 08X | Lag | 0.0325 | 0 | 0.035 |
|  |  | p | **0.002** | **0.003** | **0.006** |
|  |  | R2 | -0.27 | 0.25 | 0.24 |
| 05 | 10B | Lag | 0.04 | 0 | 0.0175 |
|  |  | p | **p < 0.001** | **0.002** | **0.03** |
|  |  | R2 | -0.41 | -0.24 | 0.17 |
| 06 | 04A | Lag | 0.0575 | 0.065 | 0.0025 |
|  |  | p | **p < 0.001** | **p < 0.001** | **p < 0.001** |
|  |  | R2 | -0.4 | -0.53 | 0.57 |
| 06 | 04B | Lag | 0 | 0 | 0 |
|  |  | p | **p < 0.001** | **p < 0.001** | **p < 0.001** |
|  |  | R2 | -0.49 | 0.32 | 0.39 |
| 09 | 07A | Lag | 0.0275 | 0 | 0.0375 |
|  |  | p | 0.081 | **p < 0.001** | **p < 0.001** |
|  |  | R2 | -0.16 | 0.39 | -0.87 |
| 09 | 07B | Lag | 0.0425 | 0 | 0 |
|  |  | p | **p < 0.001** | **p < 0.001** | **p < 0.001** |
|  |  | R2 | -0.96 | -0.78 | -0.53 |
| 10 | 06X | Lag | 0.0325 | 0 | 0.0425 |
|  |  | p | **p < 0.001** | **p < 0.001** | **p < 0.001** |
|  |  | R2 | -0.81 | 0.41 | 0.75 |
| 10 | 09A | Lag | 0.0425 | 0.0375 | 0.0475 |
|  |  | p | **p < 0.001** | **p < 0.001** | **0.004** |
|  |  | R2 | -0.36 | -0.44 | 0.27 |
| 10 | 09B | Lag | 0 | 0.0075 | 0.02 |
|  |  | p | **p < 0.001** | **p < 0.001** | **p < 0.001** |
|  |  | R2 | 0.56 | 0.49 | 0.4 |

Results from the cross-correlations for each of the indicated variable pairs. R^2^ value indicates negative and positive correlations. P-values indicating a statistically significant correlation are in boldface. All lag values are in seconds.
